# Identification of suitable methods for static pile load capacity and recommendation for BNBC 2020

**DOI:** 10.1038/s41598-026-43057-y

**Published:** 2026-04-11

**Authors:** Shakib Bhuiyan, Raisa Haque, Zahid Hasan Khan, H. M. A. Mahzuz, Shriful Islam

**Affiliations:** 1 Department of Civil Engineering, Leading University, Sylhet, 3112 Bangladesh; 2 Department of Civil and Environmental Engineering, Shahjalal University of Science and Technology, Sylhet, 3114 Bangladesh; 3 PWD Design Division 05, Public Works Department, Dhaka, Bangladesh

**Keywords:** Static pile load capacity, BNBC 2020, Cast-in-situ pile, Precast pile, SPT value, Pile load test, Solid Earth sciences, Engineering, Mathematics and computing

## Abstract

This research compares theoretical methods with static pile load test results to identify the most effective approaches for determining pile capacity across various soil and pile types, while also proposing modifications to the Bangladesh National Building Code (BNBC) 2020 guidelines. The study involved broad data collection from pile load test reports and subsoil investigations across six projects, focusing precast and cast-in-situ piles. Theoretical analyses were performed using i) bearing capacity equations as per BNBC 2020 (α-method and β-method), ii) Standard Penetration Test (SPT)-based BNBC 2020 method, and iii) SPT-based other methods from literature including Meyerhof, and Shioi Fukui equations. Following ASTM D1143 guidelines, eight evaluation methods were used to interpret load-settlement data from static load test. Statistical analyses involving Mean Absolute Percentage Error (MAPE), Bias Factor (λ), and Coefficient of Variation (COV) were conducted to evaluate predictive accuracy. Findings indicate that for clay soil, the SPT-based equations of Shioi & Fukui for skin friction and Meyerhof for end bearing can be considered as suitable alternatives for future BNBC revisions, showing lower variation with actual capacity. For sandy, silty, and layered soil devoid of clay, the SPT-based equations from BNBC 2020 are deemed appropriate.

## Introduction

The pile’s primary function is to transfer superstructure loads to deeper, less compressible, and stronger earth layers^1–3^. Piles carry loads to stable soil when near-surface conditions are insufficient, which leads to the necessity of an accurate calculation of pile bearing capacity for geotechnical design^4–6^. Pile foundations are typically utilized when the subsurface soil cannot sustain foundation loads because of either a lack of bearing capacity or the possibility of significant settling^7^. Determining pile capacity properly is a crucial task^8^. Engineering professionals assess pile capacity using a variety of accessible equations^9^. It is usually necessary to suggest a workable, trustworthy, and precise method for estimating pile bearing capacity^10,11^. Because soil parameters vary, it is hard to predict the ultimate capacity of pile foundations accurately with conservative theoretical approaches and empirical approaches, like SPT-based calculations despite improvements in instrumentation and soil knowledge^12–14^. End-bearing capacity and side resistance are the two general categories into which pile bearing capacity is divided^15^. The soil’s resistance at the pile tip provides the end-bearing capacity, while the shear parameters at the pile-soil contact creates the side resistance^16^. Shaft and base resistances are individually estimated by static analysis techniques but struggle with determining some geotechnical parameters creating questions on bearing capacity theory^17^. The critical depth concept lacks support, while dynamic testing methods provide direct results but require expertise and are limited to post-driving use^18^. The procedures involve several empirical techniques that reduce assumptions regarding soil stratigraphy, soil pile structure interaction, and soil resistance distribution next to the pile^19^.

Generally, there are two different methods to determine pile capacity:

i. Testing, such as static and dynamic load tests.
ii. Calculation, such as the pile driving formula and static design equations derived from field and laboratory research.

Over time, various methods have been employed to determine the theoretical capacity of driven piles in both cohesive and cohesionless soils using bearing capacity equations. These include:

1. Meyerhof’s Method (1976)
2. American Petroleum Institute Method (1993)
3. Tomlinson’s Method (1994)
4. Norwegian Pile Guideline (2005)
5. Indian Standard (2010)

Similarly, the following methods have been applied over the years to determine the theoretical capacity of bored piles and drilled shafts using the bearing capacity equation in both cohesive and cohesionless soil:

1. Meyerhof’s Method (1976)
2. NAVFAC DM 7.2 Method (1984)
3. AASHTO Method (1986)
4. O'Neill and Reese’s Method (1988)
5. Decourt’s Method (1995)

These methods represent a range of approaches developed and refined across several decades to evaluate the load bearing capacities of various types of piles in different soil conditions. The total side friction plus total end bearing will define the pile’s ultimate axial capacity^20^. Since accurate pile capacity calculation will lead to both cost savings and a safer construction, it should be given enough consideration.

There is still uncertainty in accurately predicting the performance of pile foundation based on design calculations, there remains a need to test piles^21,22^. To make an educated choice, a load test is therefore necessary to validate the theoretical axial capacity^23^. When the soil conditions are uncertain, pile load tests are typically carried out to collect a variety of data. In that case, test piles are built, and loads are applied prior to building the main structure’s actual load-bearing piles^24^. For the assessment of pile ultimate capacity, several scholars have proposed several approaches in the past.

The commonly used interpretations using static pile load test data are:

1. Davisson’s method (1972)
2. Brinch Hansen’s 80 percent criterion (1963)
3. Brinch Hansen’s 90 percent criterion (1963)
4. De Beer’s method (1967) or De Beer and Wallays’ method (1972)
5. Chin’s method (1970, 1971)
6. Decourt’s Extrapolation (1999)
7. Mazurkiewicz’s method (1972)
8. Fuller and Hoy’s method (1970)
9. Butler and Hoy’s method (1977)

Engineers in Bangladesh have only recently begun to employ the dynamic pile load test^25^. To find a solution of variations in pile bearing capacity assessment among different methods, the Public Works Department (PWD), Bangladesh emphasizes the challenges in pile foundation engineering. BNBC 2020 classified piles into five basic types: driven precast piles, driven cast-in-place concrete piles, prestressed concrete piles, bored piles, and drilled shaft/drilled piers^26^. Precast driven piles and cast-in-place bored piles are the most used piles. The effects of installation and the interaction between the pile and the soil affect pile load capacity^27,28^. Driven precast piles, which are displacement piles, compact the surrounding soil while being driven and acquire more resistance through reconsolidation. Conversely, cast-in-situ piles are non-displacement piles where soil removal before concreting reduces lateral stress and interface friction, especially in granular soils^29,30^.

This study aims to compare theoretical methods with static load test results for cast-in-situ and driven piles across varying soil conditions to identify the most suitable methods for static pile load capacity estimation. Additionally, it seeks to propose modifications to BNBC 2020 and integrate effective methods that are not currently specified.

Empirical pile-capacity correlations based on in situ tests are widely used, but their predictive accuracy depends on soil type, installation effects, and how adopted coefficients reflect local practice. Case-history studies show that SPT- and CPT-based approaches can exhibit different bias and scatter when evaluated against full-scale static pile load tests, motivating calibration and method selection rather than assuming a universal “best” correlation^17,18,31,32^. Within this context, the contribution of the present study is code-oriented: it evaluates the performance of BNBC 2020 provisions and selected SPT-based correlations using Bangladesh static load-test evidence and translates the findings into practical recommendations for BNBC-oriented design, including the mechanism for different bearing capacity in cohesive and cohesionless soil.

## Methodology

The general equations for calculating pile capacity are outlined in Eqs. (1–4).

General equations for pile capacity from BNBC 2020:

1 $${Q}_{ult}={Q}_{s}+{Q}_{b}-W$$

2 $${Q}_{allow}=\frac{\mathrm{Qult}}{FS}-W$$

General equations for skin friction and end bearing from BNBC 2020:

3 $${Q}_{s}={A}_{s}{f}_{s}$$

4 $${Q}_{b}={A}_{b}{f}_{b}$$

Equation (5) is used for corrections of SPT (N) value for water table (Dilatancy) in case of sandy soil for N-values with a value higher than 15.

5 $${\left(N1\right)}_{60\left(CORR\right)}=15+\frac{1}{2}\left[{\left(N1\right)}_{60}-15\right]$$

***N***_***60***_ was directly taken from the soil investigation report.

Therefore, (*N*1)_60_ = Field N (SPT) value.

The critical depth for skin friction in the case of cohesionless soil or sand is shown in Eqs. (6–8).

6 $${D}_{c}=10D \text{for loose sand}$$

7 $${D}_{c}=15D \text{for medium dense sand}$$

8 $${D}_{c}=20D \text{for dense sand}$$

The ultimate bearing capacity, which includes both skin friction and end bearing, was determined using three approaches:

(i) Static bearing capacity equations (BCE),
(ii) SPT values, and
(iii) Load tests.

The bearing capacity equations (BCE), along with SPT-based equations were taken from BNBC 2020. Apart from BNBC 2020, one method was taken from textbook and used in this research work as SPT-based other method. SPT-based other methods from literature mainly comprises Meyerhof (1976)^33^, and Shioi Fukui (1982).

To measure the deviation between theoretical and actual pile capacity, statistical analysis was employed, including Mean Absolute Percentage Error (MAPE), Bias Factor, and Coefficient of Variance (COV). These indices offer an objective approach to evaluate each method’s predictive ability and identifying trends in systemic bias or underprediction. The MAPE quantifies the average relative deviation between predicted and observed pile capacities and is defined as:

9 $${\mathrm{MAPE}}=\frac{1}{n}{\sum }_{i=1}^{n}\left|\frac{{Q}_{{\mathrm{pred}},i}-{Q}_{{\mathrm{test}},i}}{{Q}_{{\mathrm{test}},i}}\right|\times 100$$

10 $$\text{The Bias Factor }{\lambda }_{i}=\frac{{Q}_{pred,i}}{{Q}_{test,i}}$$

A mean bias factor ${\lambda }^{-}$ greater than 1.0 indicates overestimation, while a value below 1.0 denotes underestimation of pile capacity.

11 $$Coefficient\, of\, Variation\, COV=\frac{{S}_{\lambda }}{\overline{\lambda }}$$

The COV evaluates the degree of dispersion of the bias factor, where ${S}_{\lambda }$​ is the standard deviation of the bias factor. Lower COV values signify more consistent predictive performance across different projects.

## Present status of theoretical capacity of pile in BNBC 2020

The research focuses on the methods to calculate pile bearing capacity outlined in the Bangladesh National Building Code (BNBC) 2020. The BNBC has evolved to incorporate advanced research and past experiences to maintain its relevance. The BNBC was first published in 1993 to regulate building construction. While it has undergone some revisions, a comprehensive update was not conducted until 2020, resulting in the “BNBC 2020”. BNBC 2006 lacks comprehensive guidelines for determining the axial capacity of bored cast-in-situ piles, whereas the BNBC 2020 draft introduces a simplified SPT-based method to address this limitation. The methods specified in BNBC 2020 for determining the ultimate bearing capacity of a single vertical pile are as follows:

i. using static bearing capacity equations.
ii. through the application of SPT and CPT.
iii. through load tests; and
iv. through dynamic approaches.

### Physical meaning of SPT and CPT parameters used in pile-capacity correlations

#### Standard penetration test (SPT)

The measured blow count, *N*, is a dynamic penetration index affected by delivered hammer energy, borehole conditions, and effective overburden stress. For design use, *N* is commonly standardized to *N*_*60*_ by correcting the field value to an equivalent 60% energy ratio and applying procedural corrections consistent with ASTM D1586^34^. Since *N* generally increases with confining stress, many practices also normalize to a reference overburden, often expressed as (*N1*)_60_, to compare values across depth and between sites^35,36^. In SPT-based pile correlations, an averaged *N* (or *N*_*60*_) along the pile length is used as an index for shaft resistance, while an averaged value near the pile toe represents the influence zone governing end-bearing resistance. Differences among SPT methods mainly come from how the toe influence zone is defined, how the averaging is done, whether limiting values are applied in dense layers, and how cohesive and cohesionless strata are treated.

#### Cone penetration test (CPT/CPTu)

CPT provides a continuous profile of cone tip resistance (*q*_*c*_, or corrected *q*_*t*_), sleeve friction (*f*_*s*_), and, for piezocone tests, pore pressure (*u*_*2*_), consistent with ASTM D5778. Compared with discrete SPT sampling, CPT data can capture thin layers more clearly and reduce operator dependence, which is why many direct pile-capacity approaches are formulated in terms of *q*_*c*_^37,38^. In sands, *q*_*c*_ is mainly controlled by stress level and density and can be related to drained strength and stiffness. In clays, net cone resistance can be related to undrained shear strength using an empirical cone factor. Direct CPT methods estimate toe resistance from an average *q*_*c*_ over a defined zone around the pile tip and estimate shaft resistance from *q*_*c*_ along the shaft using empirical coefficients that depend on pile type and soil behavior^18^.

### Static bearing capacity equations (BCE) in BNBC 2020

Static bearing capacity equations can be used for driven precast piles as well as for driven cast-in-place piles in different soil conditions, like cohesive soil and cohesionless soil. Therefore, static bearing capacity equations (BCE) are:

- i) Bearing capacity equation in cohesive soil [α-method] for precast pile

To determine the short-term load capacity of piles installed in fine-grained soils, the α-method, which is based on total stress analysis, is typically employed^26^. As such,

12 $$Skin \, friction\, Q_{s} = f_{s} A_{s} = \alpha c_{u} A_{s}$$

13 $$End \, bearing\,Q_{b} = \, (c_{u} )_{b} (N_{c} )_{b} A_{b}$$

- ii) Bearing capacity equation in cohesionless soil [β -method] for precast pile

Both the short-term and long-term pile load capacities are determined using the β-method, which is based on an efficient stress analysis^26^. Therefore,

14 $$Skin \, friction\, f_{s} = K\sigma ^{\prime}_{z} \tan \phi \prime \, = \beta \sigma ^{\prime}_{z}$$

15 $$End \, bearing \, f_{b} = \, (\sigma ^{\prime}_{v} )_{b} (N_{q} )_{b}$$

- iii) Bearing capacity equation in cohesive soil [α-method] for cast-in-situ pile
  16 $$Skin \, friction\,Q_{s} = \, 2/3f_{s} A_{s} = \, 2/3\alpha c_{u} A_{s}$$
  17 $$End \, bearing\,Q_{b} = \, 1/3(c_{u} )_{b} (N_{c} )_{b} A_{b}$$
- iv) Bearing capacity equation in cohesionless soil [β-method] for cast-in-situ pile
  18 $$Skin \, friction\, f_{s} = \, 2/3K\sigma ^{\prime}_{z} \tan \phi \prime \, = \beta \sigma ^{\prime}_{z}$$
  19 $$End \, bearing \, f_{b} = \, 1/3(\sigma ^{\prime}_{v} )_{b} (N_{q} )_{b}$$

### SPT-based equations in BNBC 2020

The SPT-based methods can also be used for precast piles and cast-in-place piles in different soil conditions such as clayey soil, silty soil, and sandy soil. These methods are widely used in Bangladesh by practicing engineers for predictions. Following SPT-based equations are available in BNBC 2020:

- v) SPT-based BNBC 2020 method in clay soil for precast pile
  20 $$Skin \, friction:\,f_{s} = 1.8\overline{N}_{60} (inkPa) \, \le \, 70kPa$$
  21 $$End \, bearing \, f_{b} = 45N_{60} \left( {L/D} \right) \, < 4000kPa$$
- vi) SPT-based BNBC 2020 method in sandy soil for precast pile
  22 $$Skin \, friction:\,f_{s} = 2\overline{N}_{60} (inkPa) \le \, 60kPa$$
  23 $$End \, bearing \, f_{b} = 40N_{60} \left( {L/D} \right) \, \le \, 400\overline{N}_{60} < 11000kPa$$
- vii) SPT-based BNBC 2020 method in silty soil for precast pile
  24 $$Skin \, friction:\,f_{s} = 1.7\overline{N}_{60} (inkPa) \le \, 60kPa$$
  25 $$End \, bearing \, f_{b} = 30N_{60} \left( {L/D} \right) \, \le \, 300\overline{N}_{60} < 11000kPa$$
- viii) SPT-based BNBC 2020 method in clay soil for cast-in-situ pile
  26 $$Skin \, friction:\,f_{s} = 1.2\overline{N}_{60} (inkPa) \, \le \, 70kPa$$
  27 $$End \, bearing \, f_{b} = 25N_{60} \left( {L/D} \right) \, < 4000kPa$$
- xi) SPT-based BNBC 2020 method in sandy soil for cast-in-situ pile
  28 $$Skin \, friction:\,f_{s} = 1\overline{N}_{60} (inkPa) \le \, 60kPa$$
  29 $$End \, bearing \, f_{b} = 15N_{60} \left( {L/D} \right) \, \le \, 150\overline{N}_{60} < 4000kPa$$
- x) SPT-based BNBC 2020 method in silty soil for cast-in-situ pile
  30 $$Skin \, friction:\,f_{s} = 0.9\overline{N}_{60} (inkPa) \le \, 60kPa$$
  31 $$End \, bearing \, f_{b} = 10N_{60} \left( {L/D} \right) \, \le \, 100\overline{N}_{60} < 4000kPa$$

## Present status of pile load test in BNBC 2020

Verifying the pile’s actual compressive capacity in relation to its theoretical capacity is typically required for projects using pile foundations. Frequently, a static load test on the test pile is used to verify this. In general, the ultimate pile compression capacity is the load at which the pile plunges or moves rapidly with gradual or continuous increases in applied load. But often, the test does not yield a distinct plunging ultimate load. Therefore, utilizing load-settlement data from the test, a criterion must be used to calculate the pile’s ultimate capacity or failure load. Static axial compression tests on test piles are typically performed in Bangladesh in accordance with ASTM D1143^39^. It is permitted to wait at least one month following pile driving before evaluating the piles’ compressive load capacity. The steps are as follows:

- During the loading and unloading process on the test piles, load-time-settlement data is recorded.
- Test data is analyzed, presented graphically, and results are interpreted to ascertain the test piles’ ultimate and design (allowable) capability.

For pile load testing, ASTM D1143 was followed for setup, and BNBC 2020 for data interpretation. Six BNBC evaluation methods and two literature-based approaches were applied to calculate the actual pile capacity. Table 1 provides a summary of the principles and criteria of these eight interpretation methods.

i. Davisson Offset Limit,
ii. British Standard,
iii. Indian Standard,
iv. Butler-Hoy Criterion,
v. Brinch-Hansen 90% Criterion
vi. BNBC 2020 Criteria
vii. Tangent Method
viii. Terzaghi Method

**Table 1:** Summary of load test interpretation methods as per BNBC 2020.

Method	Principle / Criterion
Terzaghi (1942)	Ultimate load corresponds to settlement equal to 10% of pile diameter; allowable load is taken as 50% of that value
British Standard (BS 8004)	Allowable capacity is 50% of load causing 10% pile diameter settlement
Indian Standard (IS:2911 Part 4) / BNBC 2020	Ultimate load is the lesser of load at 10% of pile diameter or 12 mm settlement; allowable load is the smaller of two-thirds of load at 12 mm or half the load at 10% diameter settlement
Davisson Offset Limit (1973)	Defines failure where the load–settlement curve intersects an offset elastic line (0.15 in + 0.1D in); suitable for driven precast piles
Butler & Hoy (1977)	Ultimate load is at the intersection of tangents drawn to the initial linear and plunging portions of the load–settlement curve
Brinch Hansen 90% Criterion (1963)	Ultimate load corresponds to twice the settlement at 90% of full test load; allowable load uses a safety factor of 2.0–2.5
Tangent Method	Ultimate capacity found at the intersection of tangents to the initial and final segments of the load–settlement curve
BNBC 2020 Criterion	Adopts IS:2911 provisions with national calibration for settlement limits and safety factors

Each method establishes specific criteria for assessing pile load capacity, and results from theoretical methods are compared with these evaluation methods for validation. Theoretical methods often deviate from load test data, and differences continue among BNBC 2020 evaluation methods.

## SPT-based other method from textbook

Along with the BNBC 2020 correlations, two empirical SPT-based methods were applied: Meyerhof (1976) and Shioi & Fukui (1982). These approaches were chosen because of their demonstrated effectiveness in PWD projects in Bangladesh as well as their experimental reliability in cohesive and layered soils^40–42^. Therefore, both literature and field validation justify their inclusion. Equations are as followed:

- i) SPT-based other method from textbook in clay soil for precast pile
  32 $$Skin \, friction \, after \, Shioi \, Fukui \, \left( {1982} \right):f_{s} = 10N_{55} kPa$$
  33 $$End \, bearingafterMeyerhof \, \left( {1976} \right): q_{p} = Nc \, C_{u} \& q_{p} = \, 9C_{u}$$
- ii) SPT-based other method from textbook in sandy soil for precast pile
  34 $$Skin \, friction \, after \, Meyerhof \, \left( {1976} \right):fs = \, 2N_{60} \left( {kPa} \right)$$
  35 $$End \, bearingafterMeyerhof \, \left( {1976} \right):q_{p} = 40N_{60} XL/2R < \, 400N_{60} \left( {kN \, /m^{2} } \right)$$
- iii) SPT-based other method from textbook in silty soil for precast pile
  36 $$Skin \, friction \, after \, Meyerhof \, \left( {1976} \right):fs = \, 2N_{60} (kpa)$$
  37 $$End \, bearingafterMeyerhof \, \left( {1976} \right):q_{p} = 20N_{60} XL/D < \, 300N_{60} \left( {kN \, /m^{2} } \right)$$
- iv) SPT-based other method from textbook in clay soil for cast in situ pile
  38 $$Skin \, friction \, after \, Shioi \, Fukui \, \left( {1982} \right):f_{s} = 5N_{55} fPa$$
  39 $$End \, bearingafterMeyerhof \, \left( {1976} \right): q_{p} = N_{c} C_{u} \&_{ } q_{p} = \, 9C_{u}$$
- v) SPT-based other method from textbook in sandy soil for cast in situ pile
  40 $$Skin \, friction \, after \, Meyerhof \, \left( {1976} \right):f_{s} = 1N_{60} (kpa)$$
  41 $$End \, bearingafterMeyerhof \, \left( {1976} \right):q_{p} = 13N_{60} XL/2R < \, 300N_{60} \left( {kN \, /m^{2} } \right)$$
- vi) SPT-based other method from text book in silty soil for cast in situ pile
  42 $$Skin \, friction \, after \, Meyerhof \, \left( {1976} \right):fs = \, 1N_{60} (kpa)$$
  43 $$End \, bearingafterMeyerhof \, \left( {1976} \right):q_{p} = 13N_{60} XL/2R < \, 300N_{60} \left( {kN \, /m^{2} } \right)$$

## Data collection

From six projects around the country, subsurface investigation reports and related pile load test results have been gathered. Among these, four projects are from PWD, and two projects are from LGED. Among all projects, four of them are precast pile, and two of them are cast-in-situ pile. Details of the projects, including pile types, dimensions, and lengths, are provided in Table 2.

**Table 2:** Project names, types, and size of piles and pile length.

Project No	Project name and location	Pile type and size (mm)	Pile length (m)
1	Establishment of District Social Services Complex in 64 district 1st Phase, Khulna, Bangladesh	Precast and 350 X 350	17.67
2	Establishment of 40 TTC Technical Training Centre (TTC) at upazila level one Girl’s Dorm at Hobigonj Sadar Upazila, Hobigonj, Bangladesh	Precast and 305 X 305	10.67
3	Construction of 80.0 m Long PSC Girder Bridge on Khaserchar Bazar Suapur Bazar via Lalgulia Bazar Road at Ch. 200 m, Singair, Manikganj, Bangladesh	Cast-in-situ and Ø700	29.60
4	Establishment of Social Services Complex Building (64 Dist.), Barisal, Bangladesh	Precast and 350 X 350	22.25
5	Establishment of 40 TTC Technical Training Centre (TTC) at Upazila Level, Academic Building at Hobigonj Sadar Upazila, Hobigonj, Bangladesh	Precast and 356 X 356	11.59
6	Construction of 36.00 m Long RCC Girder Bridge on Patiljap Bazar Dattakanda-Charigram Hat Turning Point Road Via Kumulli Road at CH: 2400 m (Road ID NO. – 326,624,100), Upazila – Nawabganj, Dhaka, Bangladesh	Cast-in-situ and Ø600	28.00

The soil investigation data and the static pile load tests were obtained from the same project sites. Subsurface profiles, SPT-N values, and corresponding laboratory test results were collected from the geotechnical reports. These data were used directly to classify the soil layers.

**Fig. 1:**
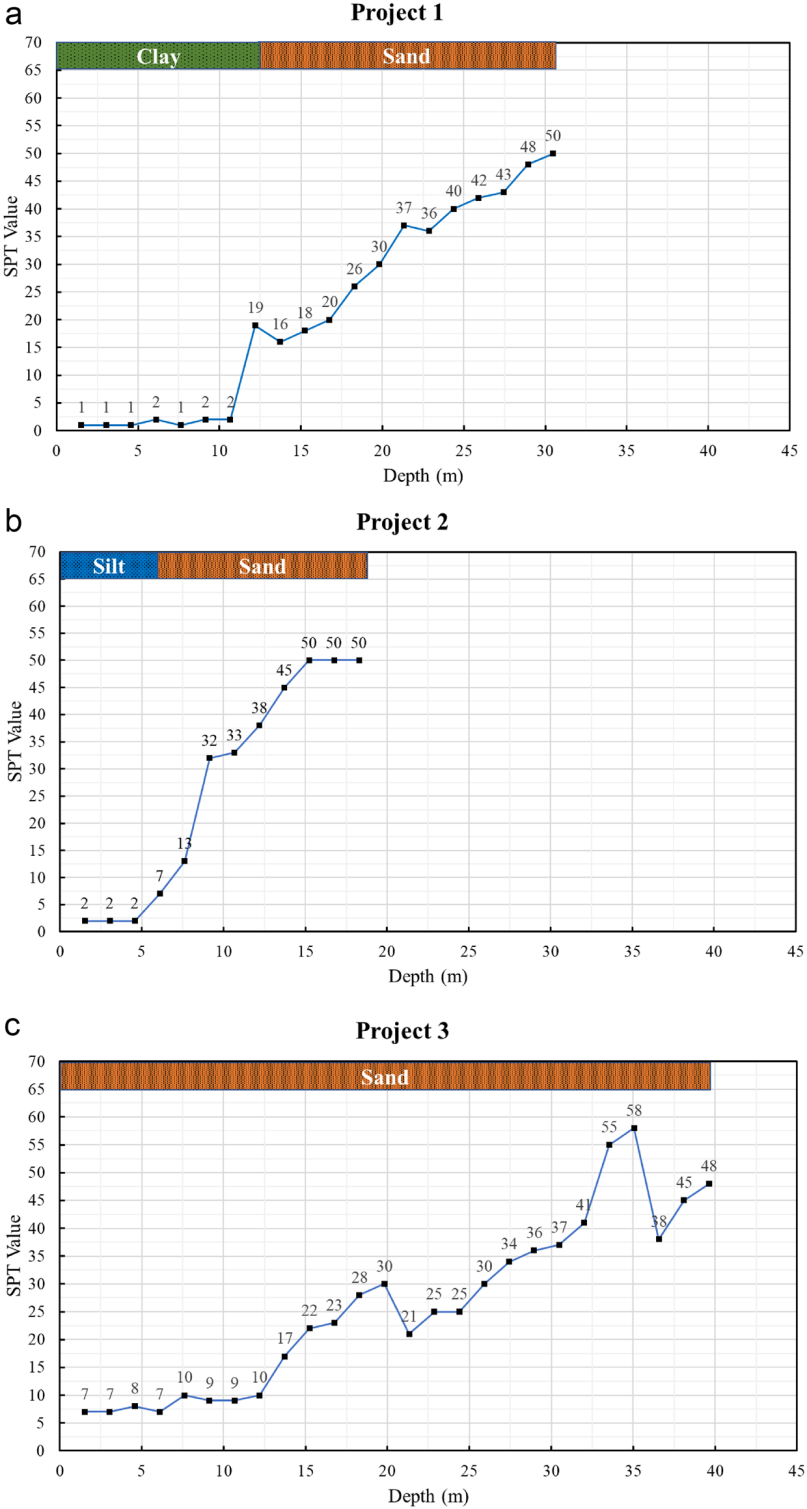

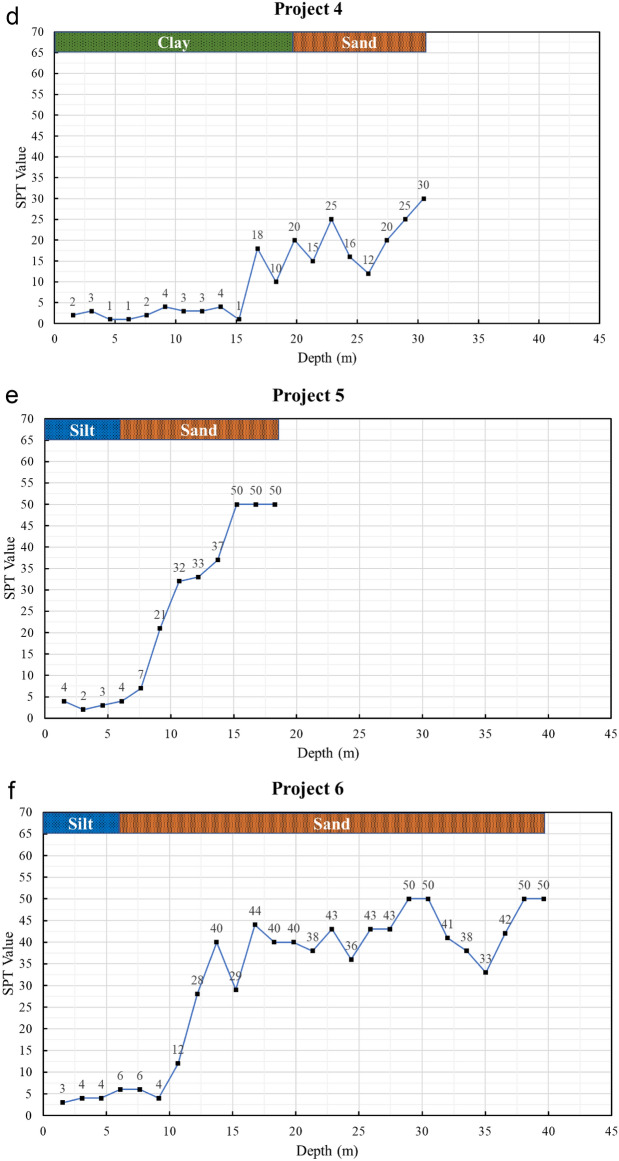
**a** SPT–depth profile for Project 1. **b** SPT–depth profile for Project 2. **c** SPT–depth profile for Project 3. **d** SPT–depth profile for Project 4. **e** SPT–depth profile for Project 5. **f** SPT–depth profile for Project 6.

The SPT data & soil profile for determining the theoretical capacity of the piles are outlined in Fig. 1a–f.

Factor of safety

- For all theoretical methods, a consistent individual factor of safety was applied: **1.5** for skin friction and **3.0** for end bearing, following the conventional practice in BNBC 2020 and Meyerhof (1976).
- In pile load test, factor of safety of **2.0** was applied for all evaluation methods as per BNBC 2020.

**Fig. 2:**
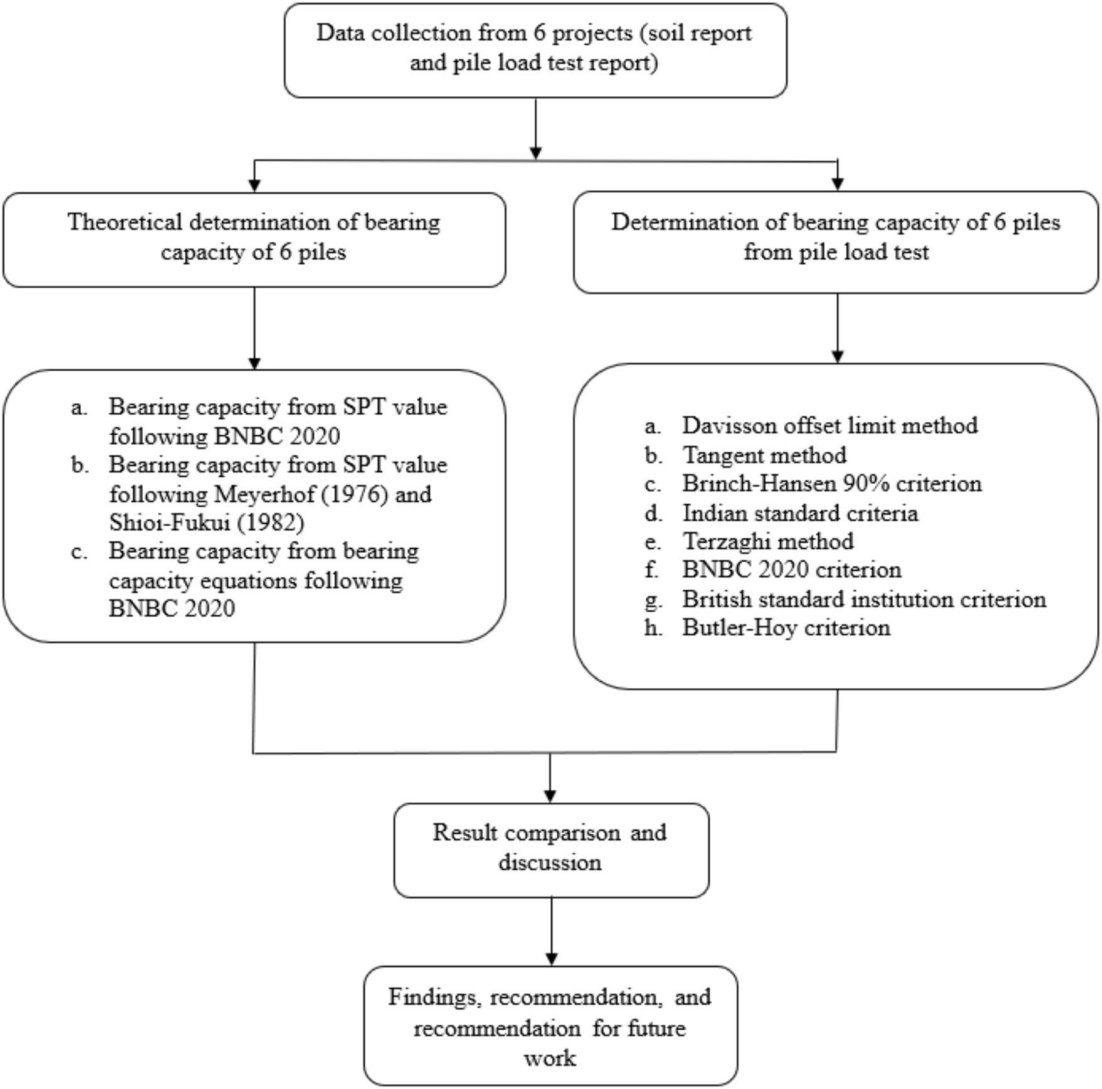
The framework of the research work.

The framework of the research work is illustrated in Fig. 2, highlighting the key methodologies and analytical approaches followed in this study.

## Results and discussion

### General overview of pile capacity calculation

**Fig. 3:**
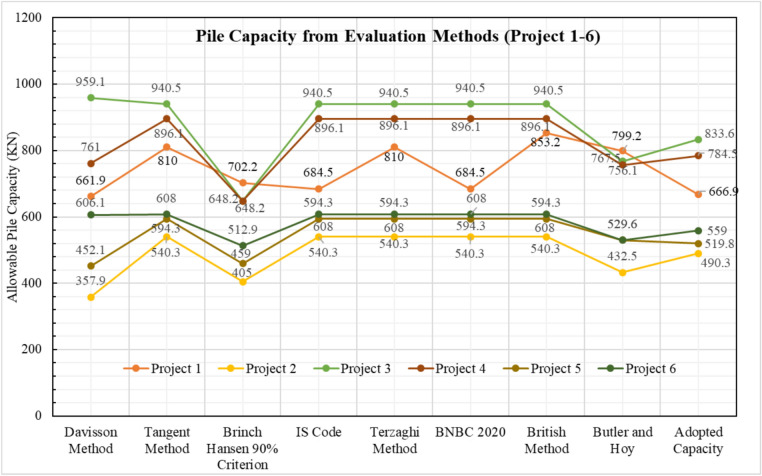
Pile capacity ($kN$) from pile load test evaluation methods.

**Fig. 4:**
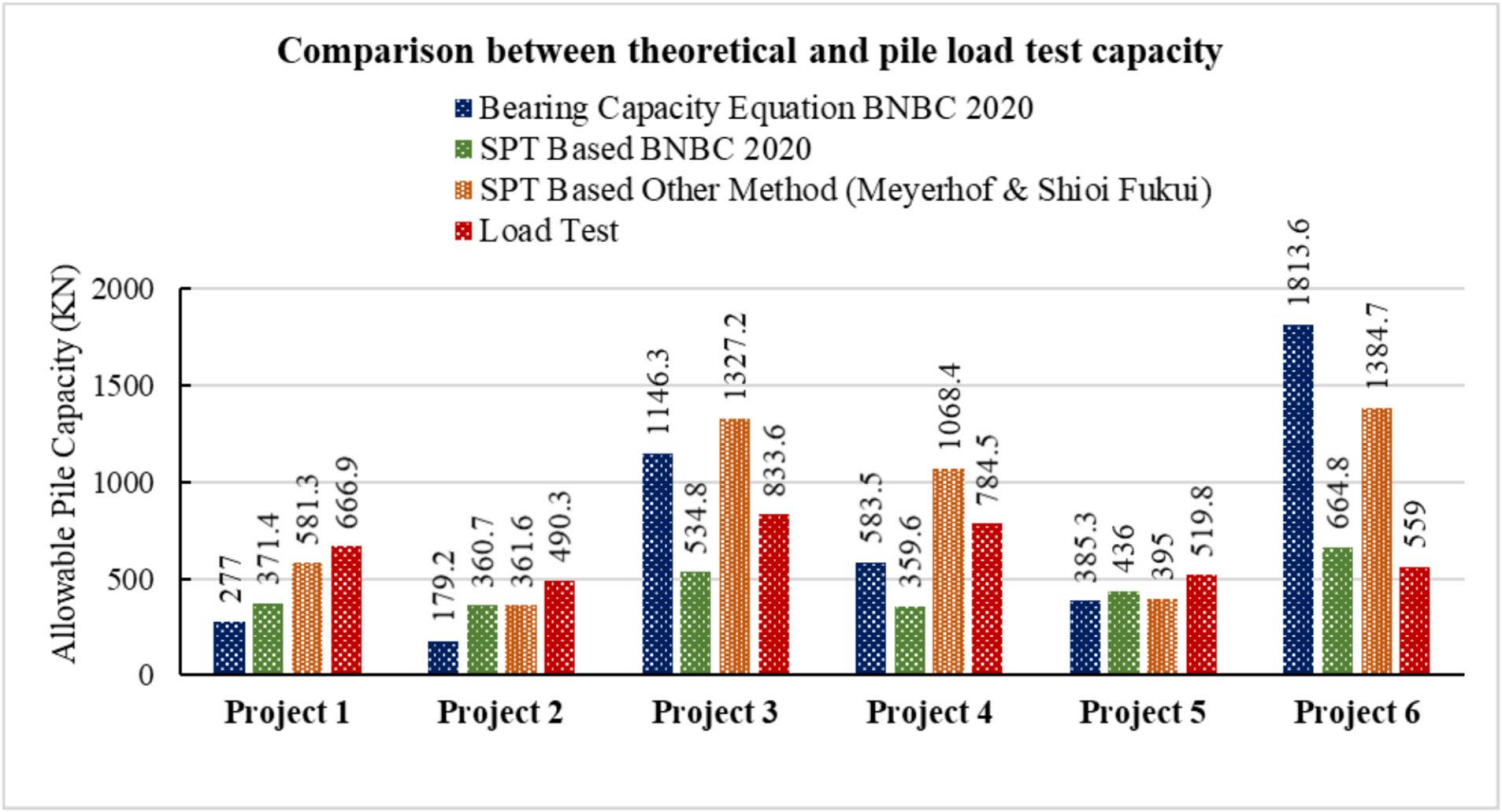
Comparison between theoretical and actual pile capacity.

Among the six projects, the soil profile of Project 3 consists entirely of sand. In contrast, the soil profile of Project 4 is predominantly clay, except for the final layer. The remaining four projects feature layered soil profiles. After performing all necessary calculations, Fig. 3 summarizes the results from the load tests conducted on test piles, including the allowable load values and the final adopted pile capacity for each case. Subsequently, Fig. 4 presents the pile capacity values derived from various theoretical methods.

### Mechanistic interpretation of method performance in cohesive and cohesionless soils

Axial pile resistance comprises shaft resistance and toe resistance, and their mobilization mechanisms differ between cohesive and cohesionless soils. In cohesive soils under short-term loading, response is commonly idealized as undrained; toe resistance depends mainly on undrained shear strength, while shaft resistance is governed by pile-soil adhesion and interface conditions. In cohesionless soils, behavior is predominantly drained and controlled by effective stresses; shaft resistance depends on horizontal effective stress and interface friction, while toe resistance depends on the shearing resistance beneath the pile tip. Installation effects can modify these components by changing radial stresses and disturbance around the shaft and toe, which is particularly relevant when comparing driven precast piles with bored cast-in-situ piles^43,44^.

These mechanisms help explain the trends observed in the six case histories. In clay-dominant profiles (Projects 1 and 4), differences among methods are mainly driven by how unit shaft resistance is inferred in clay layers, which leads to larger underestimation or scatter when conservative conversions from SPT indices are used.

In sand and sand-silt profiles (Projects 2, 3, 5, and 6), closer agreement is generally expected because SPT indices correlate more directly with drained behavior; however, overprediction can occur in dense strata when methods do not adequately control limiting resistance at high *N* values.

### Evaluation of pile load test interpretation methods

**Fig. 5:**
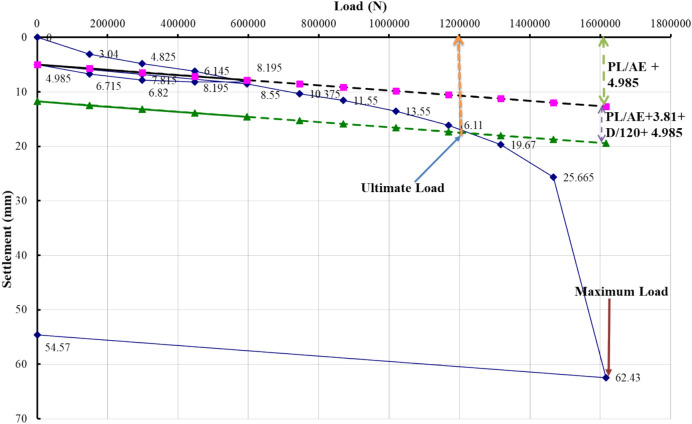
Load–settlement curve interpreted using Davisson Offset Limit Method.

**Fig. 6:**
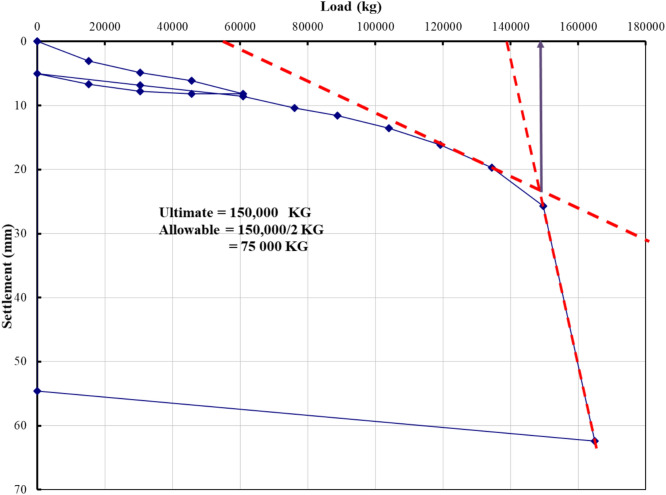
Load–settlement curve interpreted using Tangent Method.

**Fig. 7:**
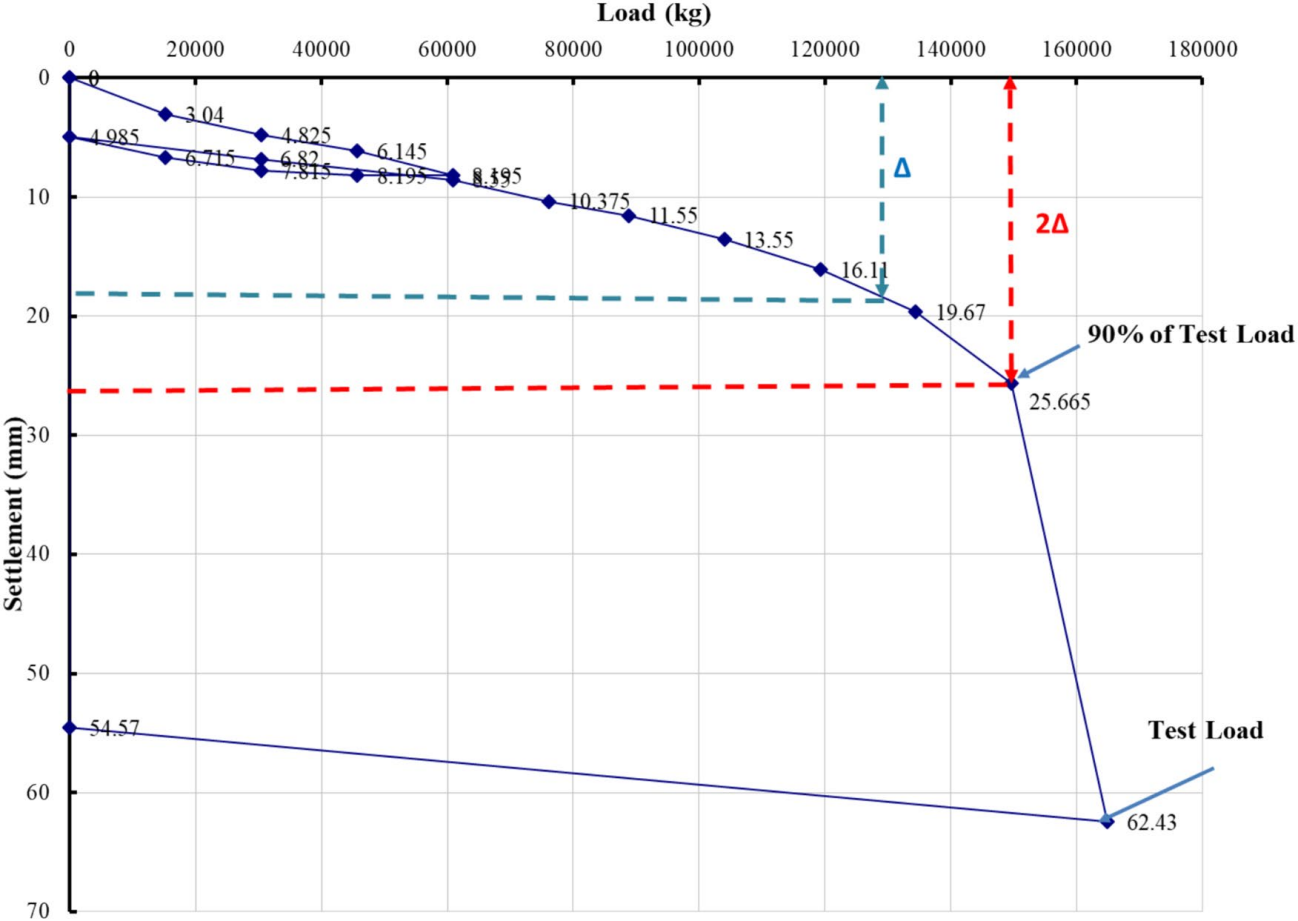
Load–settlement curve interpreted using Brinch Hansen 90% Criterion.

**Fig. 8:**
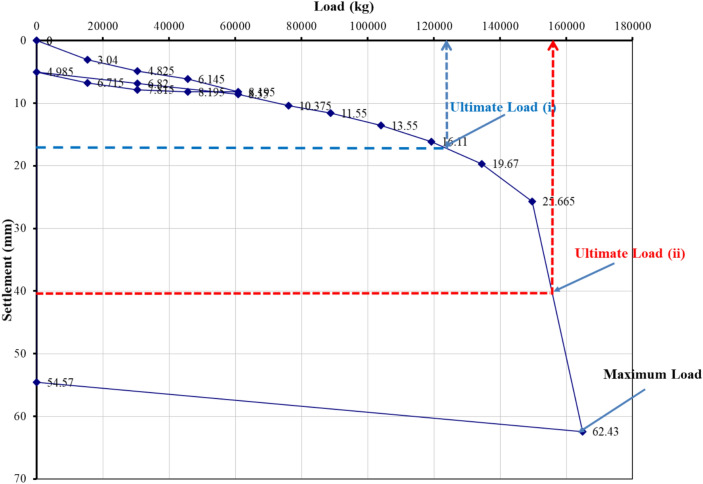
Load–settlement curve interpreted using IS Code Criterion.

**Fig. 9:**
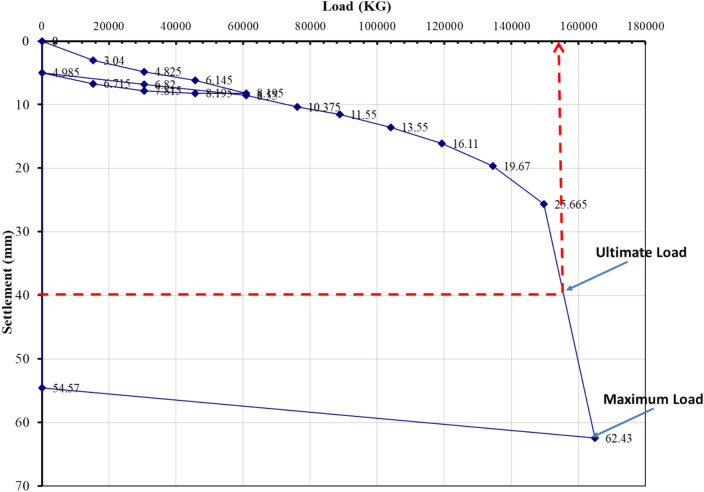
Load–settlement curve interpreted using Terzaghi Method.

**Fig. 10:**
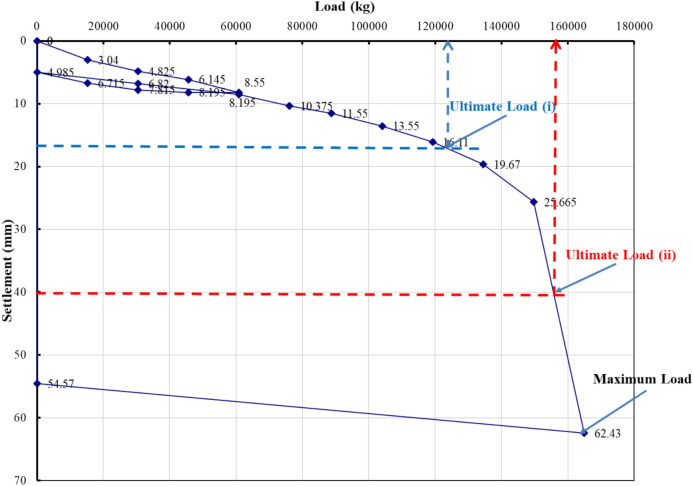
Load–settlement curve interpreted using BNBC 2020 Criterion.

**Fig. 11:**
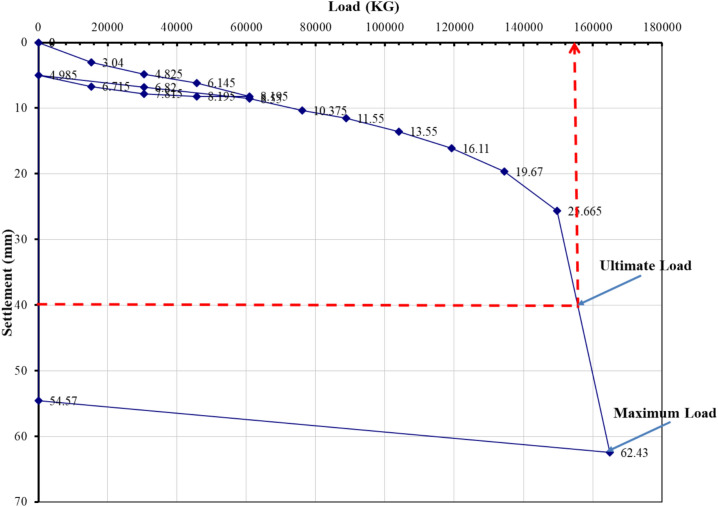
Load–settlement curve interpreted using British Standard Method.

**Fig. 12:**
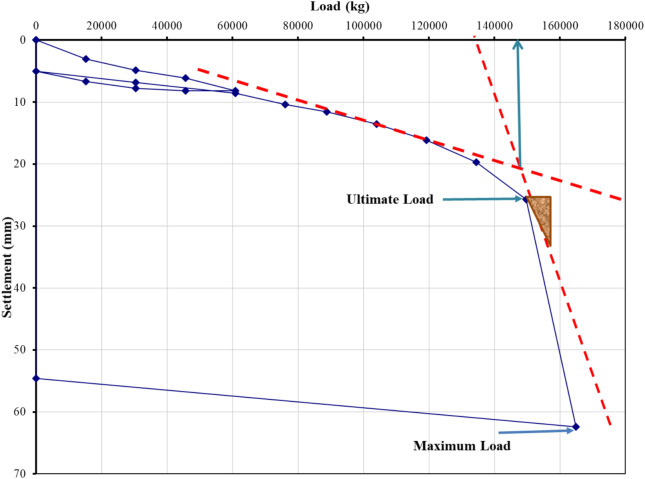
Load–settlement curve interpreted using Butler and Hoy Criterion.

The load–settlement behavior of Project 1 was interpreted using eight evaluation methods recognized in BNBC 2020 and related international standards. Figures 5, 6, 7, 8, 9, 10, 11, 12 illustrate the graphical interpretation of each method, showing both the ultimate and allowable load capacities derived from the field test. The detailed calculations are summarized in the following subsections.

#### Davisson offset limit method

Ultimate capacity = 1,200,000 N = 122,449 kg.

Allowable capacity = 122,449 / 2 = 61,224 kg = 661.9 kN.

#### Tangent method

Ultimate capacity = 150,000 kg.

Allowable capacity = 150,000 / 2 = 75,000 kg = 810.0 kN.

#### Brinch Hansen 90% criterion

Test load = 164,905 kg.

90% of test load = 148,414 kg.

2Δ = 26 mm → Δ ≈ 18 mm.

Corresponding test load = 130,000 kg.

Ultimate capacity = 130,000 kg.

Allowable capacity = 130,000 / 2 = 65,000 kg = 702.2 kN.

#### IS code criterion

D = 14 in → 10% of D + 4.985 = 40 mm.

Ultimate / final capacity (minimum of):

i. Final load at 12 mm + 4.985 settlement = 121,000 kg
ii. Load corresponding to 10% of D = 155,000 kg

Allowable capacity (minimum of):

i. 2/3 × 121,000 = 63,333 kg
ii. 1/2 × 155,000 = 77,500 kg

The minimum of the two criteria is considered as the allowable capacity for analysis, corresponding to the plotted allowable value of 684.5 kN.

#### Terzaghi method

D = 14 in → 10% of D + 4.985 = 40 mm.

Ultimate capacity = 158,000 kg.

Allowable capacity = 158,000 / 2 = 79,000 kg = 810.0 kN.

#### BNBC 2020 criterion

D = 14 in → 10% of D + 4.985 = 40 mm.

Ultimate / final capacity (minimum of):

i. Final load at 12 mm + 4.985 settlement = 121,000 kg.
ii. Load corresponding to 10% of D = 155,000 kg.

Allowable capacity (minimum of):

i. 2/3 × 121,000 = 63,333 kg
ii. 1/2 × 155,000 = 77,500 kg

Allowable capacity = 63,333 kg = 684.5 kN.

#### British standard method

D = 14 in → 10% of D + 4.985 = 40 mm.

Ultimate capacity = 158,000 kg.

Allowable capacity = 158,000 / 2 = 79,000 kg = 853.2 kN.

#### Butler and Hoy criterion

Tangent drawn to initial portion and tangent to plunging portion with slope 1 in/20 ton (≈ 1 kN/0.14 mm).

Ultimate capacity = 148,000 kg.

Allowable capacity = 148,000 / 2 = 74,000 kg = 799.2 kN.

The interpreted allowable capacities range between 661.9 kN and 853.2 kN, reflecting the influence of differing failure criteria. The field-adopted capacity of 666.9 kN was empirically selected within this range, representing a practical midpoint rather than a value derived from any specific interpretation method. Complete load–settlement plots and corresponding analyses for Projects 2–6 are presented in the appendix.

### Project 1: Social services complex, Khulna, building office building

**Fig. 13:**
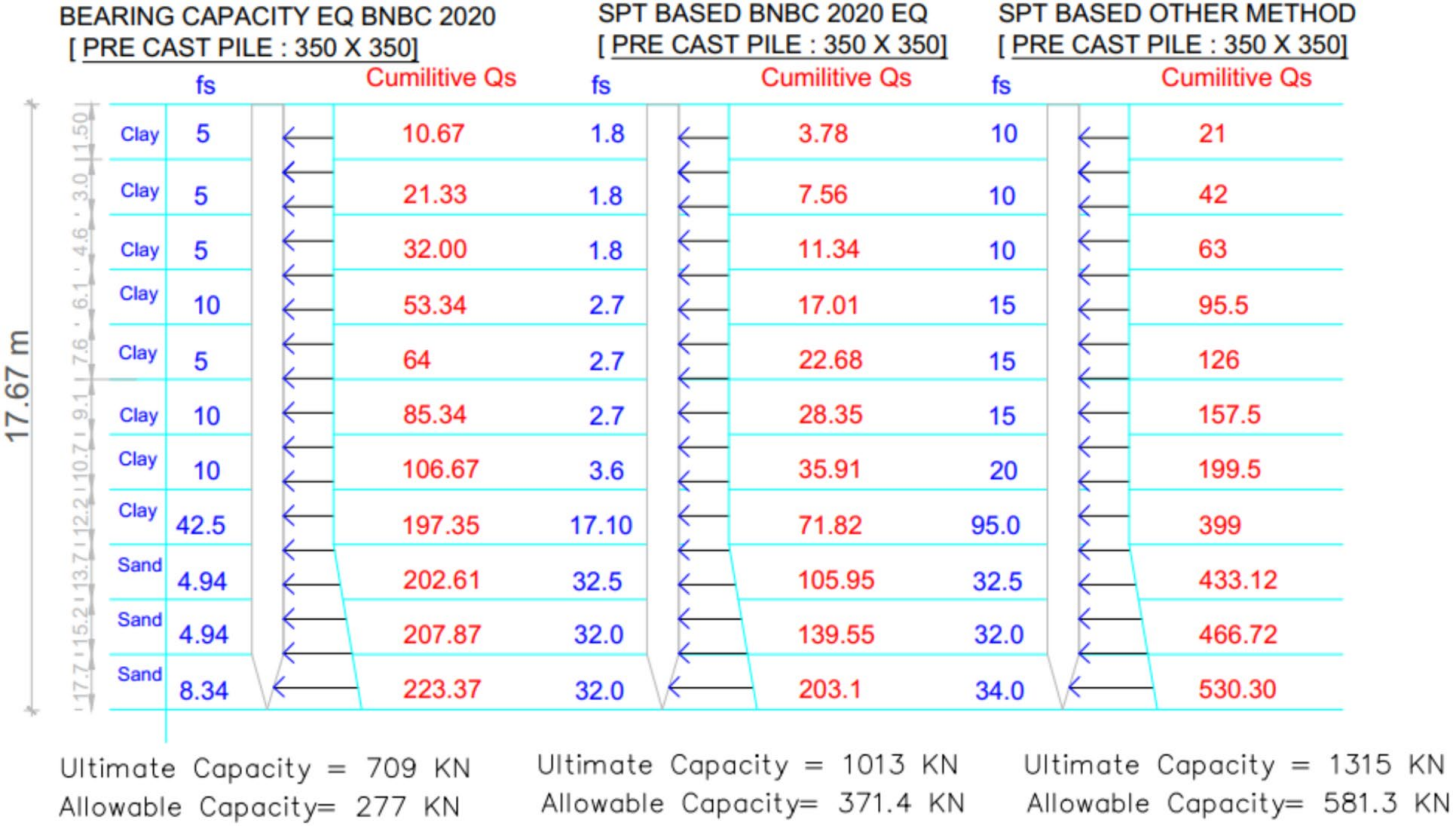
Variation of theoretical capacity in three methods for Project 1.

The soil profile of Project 1 primarily consists of clay with several sand layers at the base. Figure 13 illustrates the changes in skin-friction resistance along depth and the variation of theoretical pile capacities obtained from the three theoretical methods bearing capacity equation BNBC 2020, SPT-based BNBC 2020, and the SPT-based other method.

Table 3 presents a detailed statistical comparison between the theoretical capacities and the measured static load-test result of 666.9 kN. The SPT-based BNBC 2020 method produced an allowable capacity of 371.4 kN, corresponding to a deviation of − 44.31% from the load-test value and a bias factor (λ) of 0.56. This underestimation is mainly attributed to the reduced cumulative skin-friction component (71.82 kN) obtained from this approach. In contrast, the SPT-based other method following Meyerhof (1976) and Shioi & Fukui (1982) estimated an allowable capacity of 581.3 kN, yielding a deviation of − 12.82% and a bias factor of 0.87, which shows the closest agreement with the measured result. The higher accuracy of this method results from greater cumulative skin friction (399 kN) and end-bearing resistance (530.3 $kN$). Alternatively, the bearing-capacity equation from BNBC 2020 predicted a considerably lower allowable capacity of 277.0 kN (− 58.46%), with a corresponding bias factor of 0.42.

**Table 3:** Statistical comparison of theoretical and load-test capacities for Project 1.

Method	Soil type	Cum. skin friction ($kN$)	Skin friction, *Q*_*s*_ ($kN$)	End bearing, *Q*_*b*_ ($kN$)	Allowable capacity ($kN$)	Difference with load test (666.9 $kN$)	Bias factor, λ = Pred/Test	Statistical summary
SPT-based BNBC 2020	Clay	71.82	203.1	861	371.4	– 44.31%	0.56	MAPE = 38.54%, Mean bias, λ̄ = 0.61 & COV = 0.31
	Sand	131.28						
SPT-based Other	Clay	399	530.3	836.4	581.3	– 12.82%	0.87	
	Sand	131.31						
BCE BNBC 2020	Clay	197.35	223.37	537.27	277.0	– 58.46%	0.42	
	Sand	26.02						

*Pred* Predicted capacity, *Test* Actual capacity from load test.

**Fig. 14:**
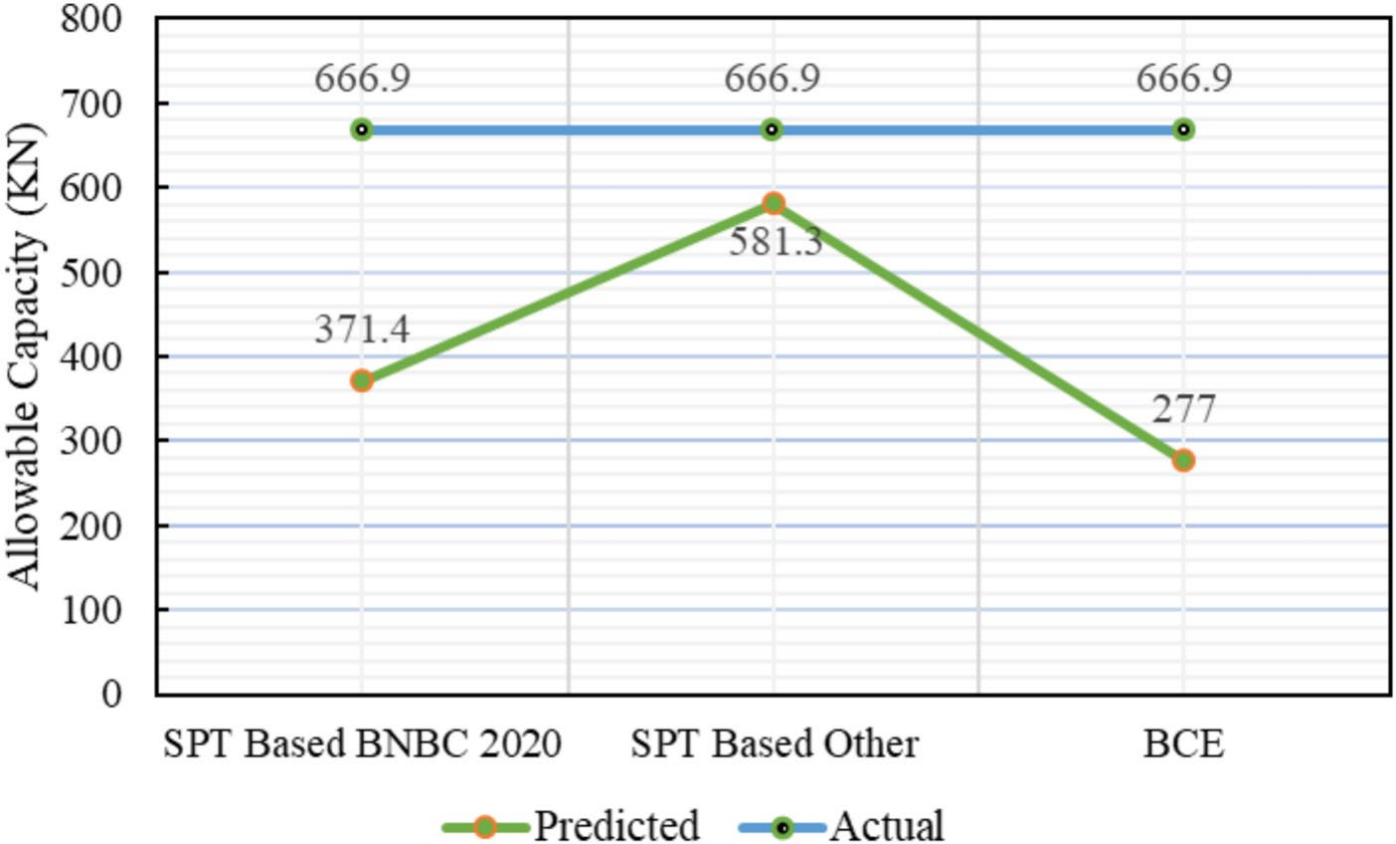
Deviations between predicted and actual pile capacities for Project 1.

Figure 14 summarizes the deviations between the predicted and actual pile capacities for all three methods. The statistical evaluation indicates a mean bias (λ̄) of 0.61, MAPE = 38.54%, and COV = 0.31, reflecting a general underprediction but moderate variability among theoretical methods. Overall, the SPT-based other methods demonstrated superior reliability for soil composed of both clay and sand layers, closely matching the measured pile capacity.

### Project 2: Project -40 TTC (Technical Training Centre), Hobigonj, Girl’s Dorm

**Fig. 15:**
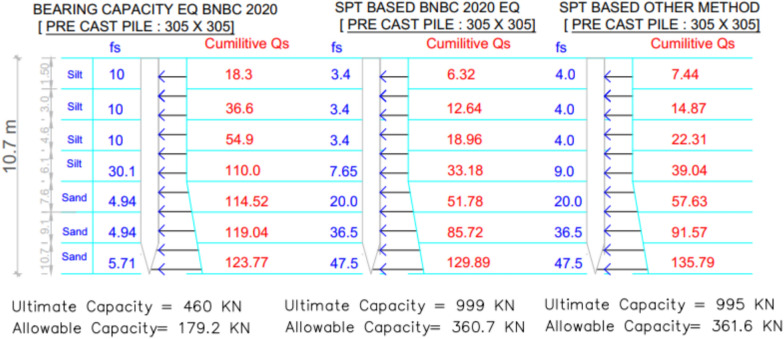
Variation of theoretical capacity in three methods for Project 2.

The analysis for Project 2 highlights the variations in predicted pile capacities among the three theoretical methods, as illustrated in Fig. 15, where changes in skin-friction distribution along depth are also presented. The subsurface profile predominantly consists of layered silt and sand, influencing both shaft resistance and end-bearing behavior.

A comparative summary between theoretical predictions and the measured pile load-test result (490.3 kN) is presented in Table 4. The SPT-based BNBC 2020 method calculated an allowable capacity of 360.7 kN, corresponding to a deviation of − 26.43% and a bias factor (λ) of 0.74. Similarly, the SPT-based other methods, following Meyerhof (1976) and Shioi & Fukui (1982), estimated a capacity of 361.6 kN, showing a deviation of − 26.26% and an identical bias factor of 0.74. Both approaches demonstrate comparable accuracy and indicate satisfactory performance in cohesionless soil conditions. Conversely, the BNBC 2020 bearing-capacity equation (BCE) measured a considerably lower estimate of 179.2 kN, reflecting an underprediction of − 63.46% with a bias factor of 0.37, which confirms its conservative tendency for sandy-silty strata.

**Table 4:** Statistical comparison of theoretical and load-test capacities for Project 2.

Method	Soil type	Cum. skin friction (kN)	Skin friction, *Q*_*s*_ (kN)	End bearing, *Q*_*b*_ (kN)	Allowable capacity (kN)	Difference with load test (490.3 kN)	Bias factor, λ = Pred/Test	Statistical summary
SPT-based BNBC 2020	Silt	33.18	129.89	892.8	360.7	– 26.43%	0.74	MAPE = 38.71%, Mean bias, λ̄ = 0.61 & COV = 0.29
	Sand	96.71						
SPT-based Other	Silt	39.04	135.79	883.5	361.6	– 26.26%	0.74	
	Sand	96.75						
BCE BNBC 2020	Silt	110.00	123.77	360.27	179.2	– 63.46%	0.37	
	Sand	13.77						

**Fig. 16:**
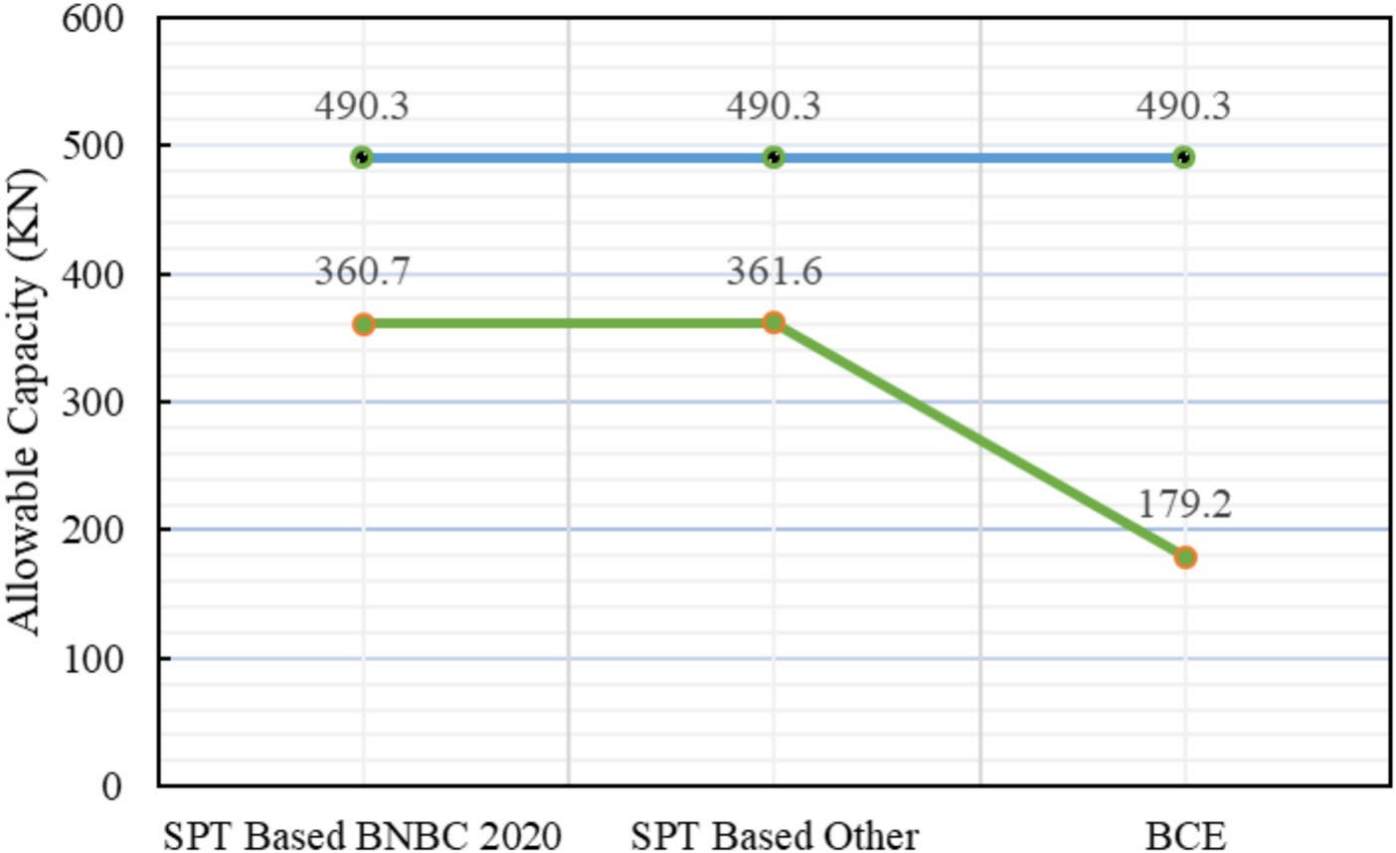
Deviations between predicted and actual pile capacities for Project 2.

The statistical evaluation yielded a mean bias (λ̄) of 0.61, MAPE = 38.71%, and COV = 0.29, signifying moderate variability among the predictive methods. The deviation trends are summarized in Fig. 16, which clearly reflects the relative performance of each analytical approach against the measured load-test capacity. Among these, the SPT-based BNBC 2020 and the other methods provided the most consistent predictions for the silt–sand layered deposit.

### Project 3: Construction of 80.0m long PSC Girder Bridge at Manikganj

**Fig. 17:**
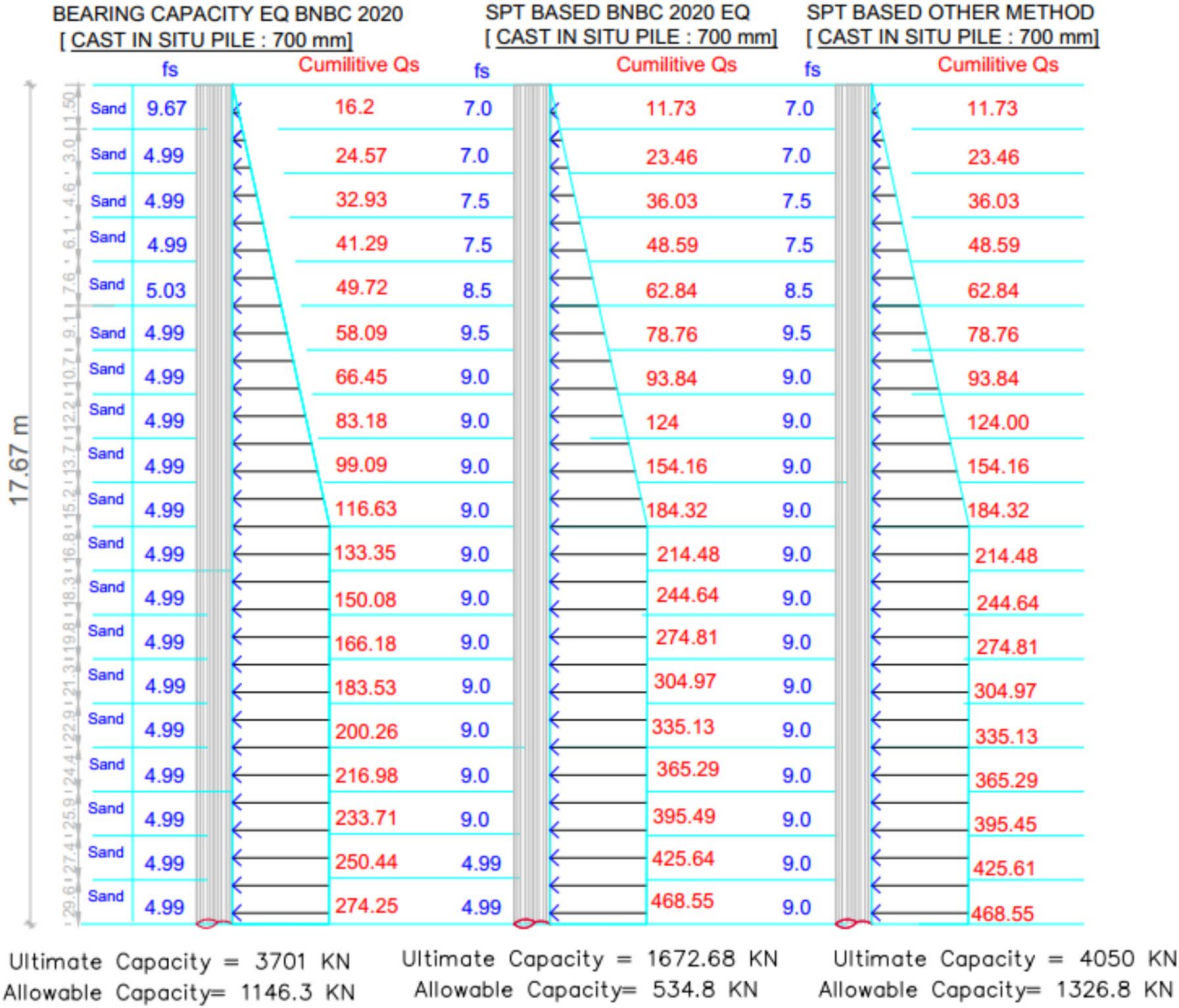
Variation of theoretical capacity in three methods for Project 3.

For Project 3, Fig. 17 illustrates the variation in skin friction values obtained from the three theoretical methods. The site investigation confirmed that the subsoil profile is entirely composed of sand. Table 5 presents a statistical comparison between the theoretical predictions and the load test result, which confirmed an allowable pile capacity of 833.6 kN.

**Table 5:** Statistical comparison of theoretical and load-test capacities for Project 3.

Method	Soil type	Cum. skin friction (kN)	Skin friction, *Q*_*s*_ (kN)	End bearing, *Q*_*b*_ (kN)	Allowable capacity (kN)	Difference with load test (833.6 kN)	Bias factor, λ = Pred/Test	Statistical summary
SPT-based BNBC 2020	Clay	NA	468.55	1472.6	534.8	– 35.85%	0.64	**MAPE** = 44.19%, Mean bias, λ̄ = 1.20 & COV = 0.34
	Sand	468.55						
SPT-based Other	Clay	NA	468.55	3850	1327.2	+ 59.22%	1.59	
	Sand	468.55						
BCE BNBC 2020	Clay	NA	274.25	3696	1146.3	+ 37.52%	1.38	
	Sand	274.25						

**Fig. 18:**
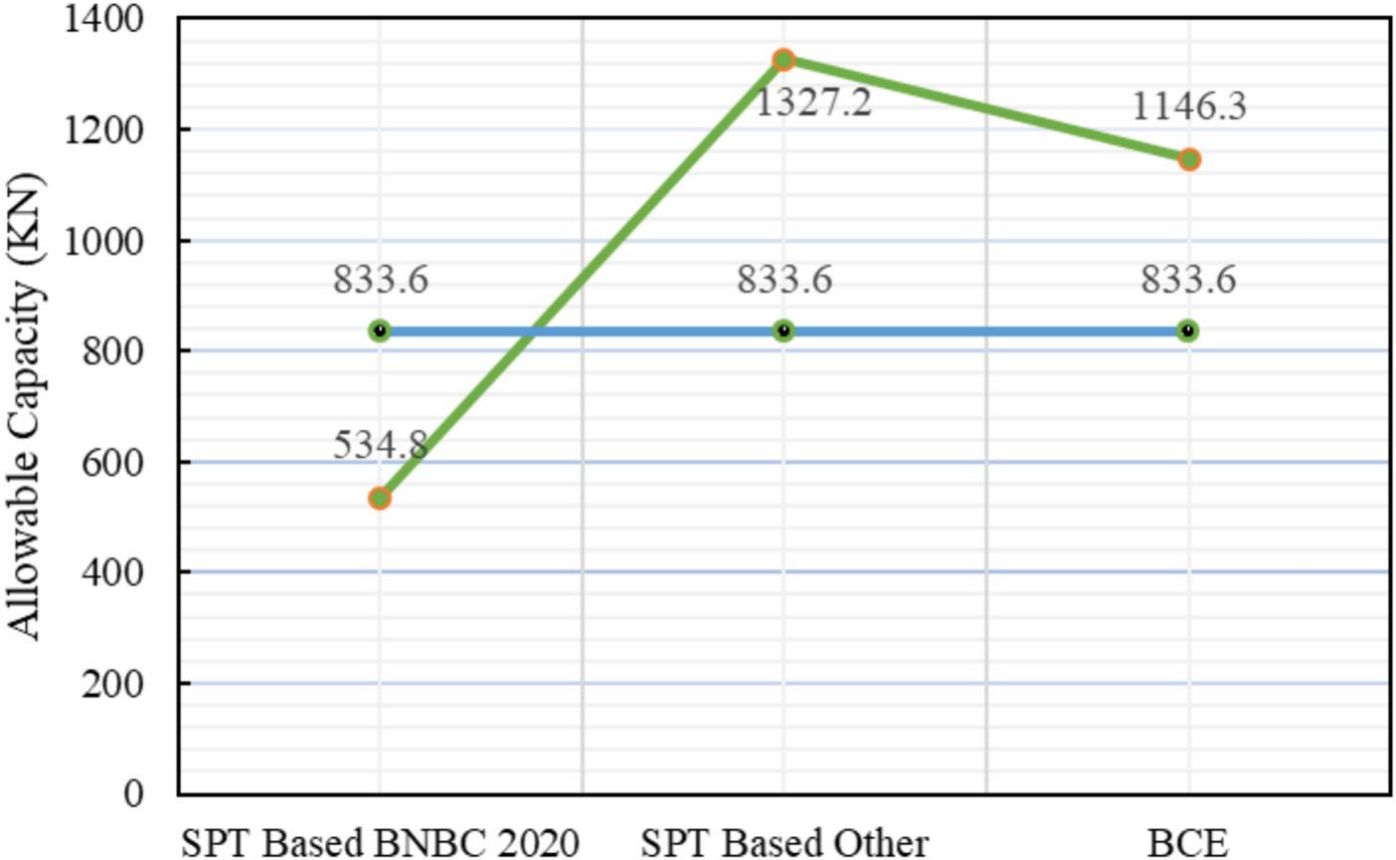
Deviations between predicted and actual pile capacities for Project 3.

The SPT-based BNBC 2020 method estimated the capacity at 534.8 kN, indicating a deviation of – 35.85% from the load test result. In contrast, the SPT-based other methods predicted a capacity of 1327.2 kN, corresponding to a deviation of + 59.22%. The bearing capacity equation BNBC 2020 estimated a capacity of 1146.3 $kN$, which is + 37.52% higher than the load test result. Statistical analysis shows that the SPT-based BNBC 2020 method yields a mean bias (λ̄) of 1.20, a coefficient of variation (COV) of 0.34, and a MAPE value of 44.19%, reflecting more consistent predictions under sandy soil conditions. On the other hand, both the SPT-based other methods and the bearing capacity equation of BNBC 2020 exhibited comparatively higher bias values, indicating that the SPT-based BNBC 2020 method provides more practical accuracy for uniform sandy soils. As shown in Fig. 18, the deviations between the predicted and actual pile capacities further confirm the consistent performance of the SPT-based BNBC 2020 approach.

### Project 4: Establishment of social services complex building, Barisal, Bangladesh

**Fig. 19:**
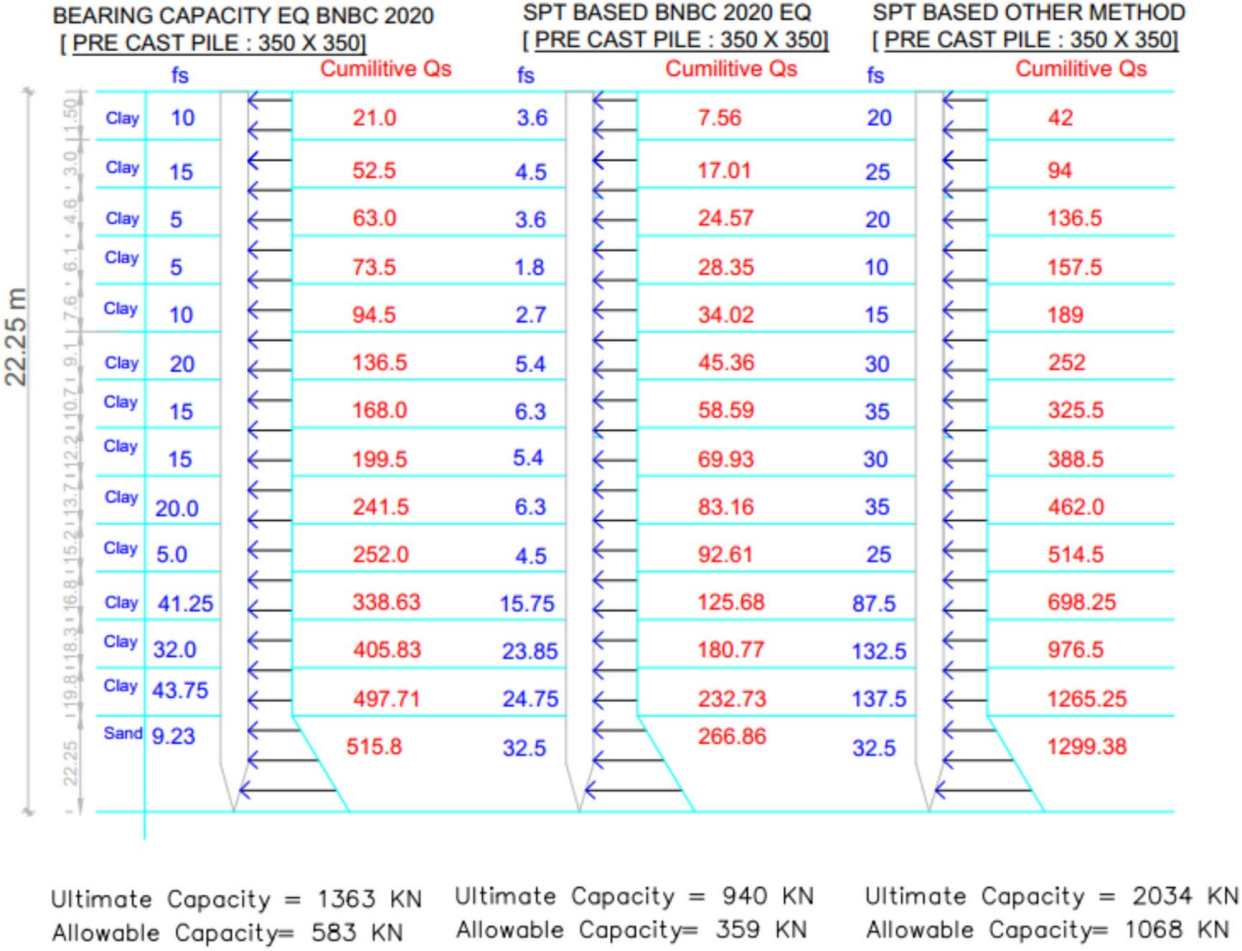
Variation of theoretical capacity in three methods for Project 4.

For Project 4, the variation in skin friction obtained from the three theoretical methods is displayed in Fig. 19. The subsoil profile predominantly consists of clay with a thin sand layer at the base. As depicted in Table 6, the pile load test confirmed an allowable capacity of 784.5 $kN$. The SPT-based BNBC 2020 method predicted a capacity of 359.6 kN, corresponding to a deviation of -54.16% from the load test result. In contrast, the SPT-based other methods measured a capacity of 1068.4 kN, representing a positive discrepancy of + 36.19%. The bearing capacity equation BNBC 2020 estimated a capacity of 583.5 kN, which is 25.62% lower than the test value. The SPT-based BNBC 2020 method produced a total skin friction of 266.86 kN (232.73 kN in clay and 34.13 kN in sand), whereas the SPT-based other methods calculated a much higher total of 1299.38 kN (1265.3 kN in clay and 34.13 kN in sand). The bearing capacity equation BNBC 2020 obtained 515.8 kN of total skin friction (497.71 kN in clay and 18.09 kN in sand). The comparatively lower capacity derived from the SPT-based BNBC 2020 method is attributed to its smaller skin friction component in cohesive soil.

**Table 6:** Statistical comparison of theoretical and load-test capacities for Project 4.

Method	Soil type	Cum. skin friction (kN)	Skin friction, *Q*_*s*_ (kN)	End bearing, *Q*_*b*_ (kN)	Allowable capacity (kN)	Difference with load test (784.5 kN)	Bias factor, λ = Pred/Test	Statistical summary
SPT-based BNBC 2020	Clay	232.73	266.86	738	359.6	– 54.16%	0.46	MAPE = 38.66%, Mean bias, λ̄ = 0.85 & COV = 0.44
	Sand	34.13						
SPT-based Other	Clay	1265.3	1299.38	799.5	1068.4	+ 36.19%	1.36	
	Sand	34.13						
BCE BNBC 2020	Clay	497.71	515.8	911.94	583.5	– 25.62%	0.74	
	Sand	18.09						

**Fig. 20:**
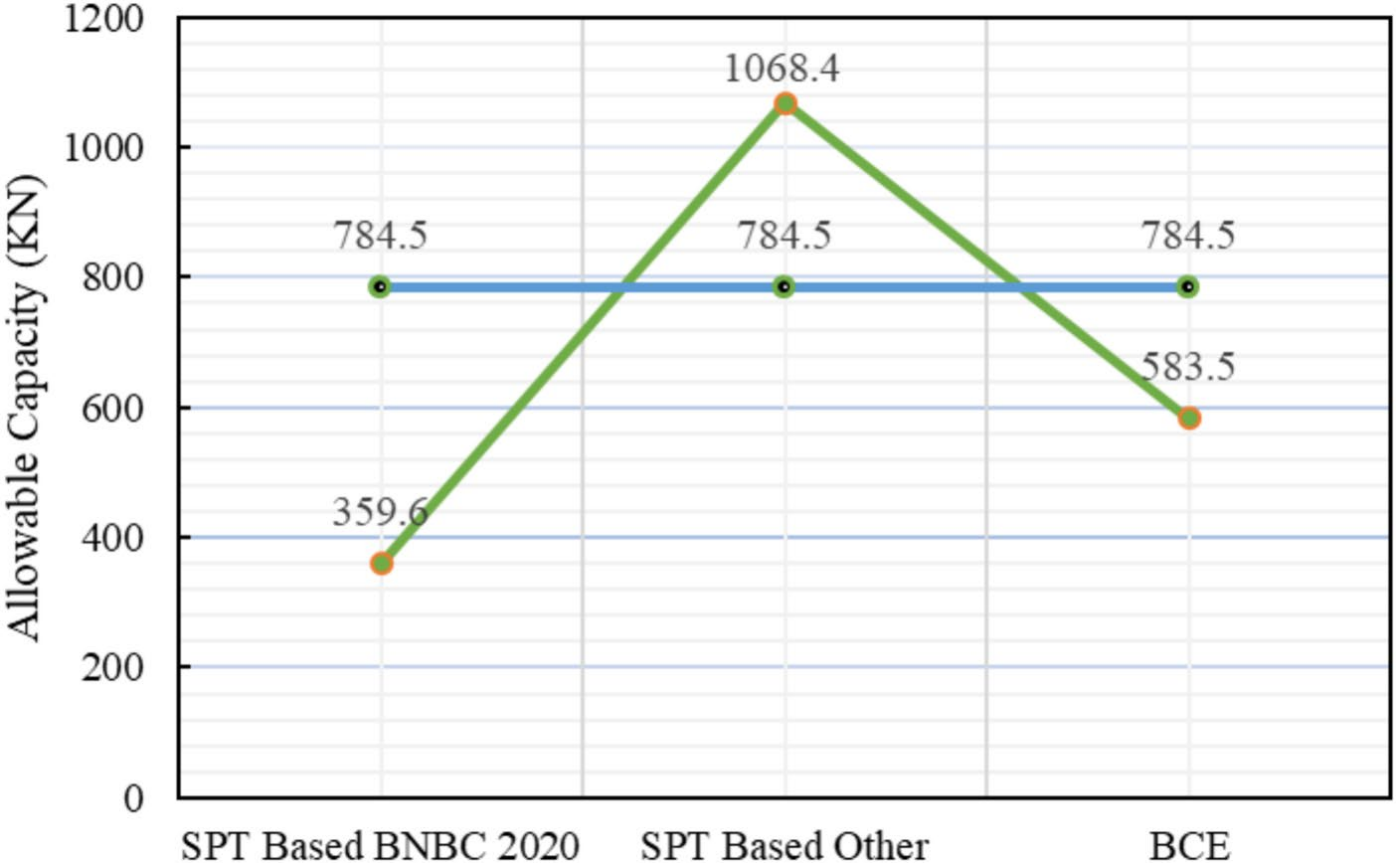
Deviations between predicted and actual pile capacities for Project 4.

Statistical analysis shows that the mean bias (λ̄) of 0.85, a COV of 0.44, and a MAPE of 38.66%, reflecting conservative yet consistent predictions. Both the SPT-based other methods and the bearing capacity equation BNBC 2020 displayed higher bias values, indicating greater variability in their estimates. As shown in Fig. 20, the deviations between the predicted and actual pile capacities further confirm the relatively reliable performance of the SPT-based BNBC 2020 method for predominantly clayey soil conditions.

### Project 5: 40 TTC (Technical Training Centre), Hobigonj, Academic Building

**Fig. 21:**
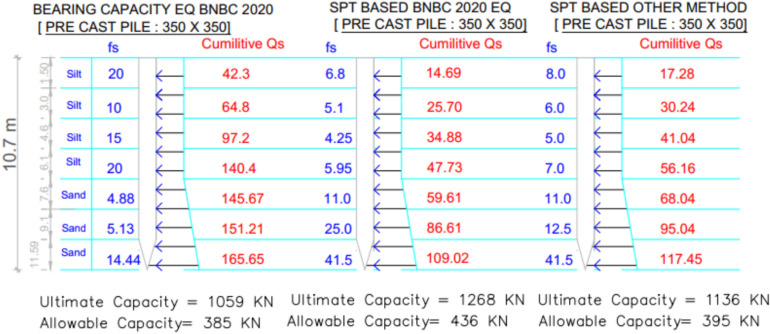
Variation of theoretical capacity in three methods for Project 5.

The subsoil investigation for Project 5 identified a stratified profile of sand and silt. Figure 21 portrays the skin friction outcomes across the theoretical methods. As shown in Table 7, the pile load test confirmed an allowable capacity of 519.8 kN. The SPT-based BNBC 2020 method predicted 436.0 kN, corresponding to a 16.12% lower value than the measured capacity. The SPT-based other methods produced a capacity of 395.0 kN, showing a 24.0% reduction. Meanwhile, the bearing capacity equation BNBC 2020 provided the most conservative estimate of 385.3 kN, differing by 25.88% from the test results.

**Table 7:** Statistical comparison of theoretical and load-test capacities for Project 5.

Method	Soil type	Cum. skin friction (kN)	Skin friction, *Q*_*s*_ (kN)	End bearing, *Q*_*b*_ (kN)	Allowable capacity (kN)	Difference with load test (519.8 kN)	Bias factor, λ = Pred/Test	Statistical summary
SPT-based BNBC 2020	Silt	47.736	109.026	1193.8	436.0	– 16.12%	0.84	MAPE = 22.00%, Mean bias, λ̄ = 0.78 & COV = 0.05
	Sand	61.29						
SPT-based Other	Silt	56.16	117.45	1054.1	395.0	– 24%	0.76	
	Sand	61.29						
BCE BNBC 2020	Silt	140.4	165.65	928.38	385.3	– 25.88%	0.74	
	Sand	25.25						

**Fig. 22:**
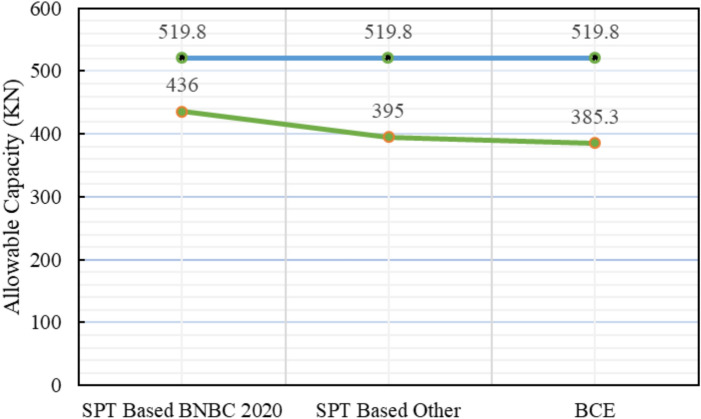
Deviations between predicted and actual pile capacities for Project 5.

Statistical analysis further supports this observation, with a mean bias (λ̄) of 0.78, a COV of 0.05, and a MAPE of 22.00%, indicating consistent and dependable predictions. In contrast, both the SPT-based other methods and the bearing capacity equation BNBC 2020 demonstrated slightly larger discrepancies. Figure 22 reveals the comparison between theoretical predictions and actual capacities that underscores the steady performance of the SPT-based BNBC 2020 method for stratified sand–silt conditions.

### Project 6: 36.0 m long RCC Girder Bridge, Dhaka [cast-in-situ pile Ø 22″]

**Fig. 23:**
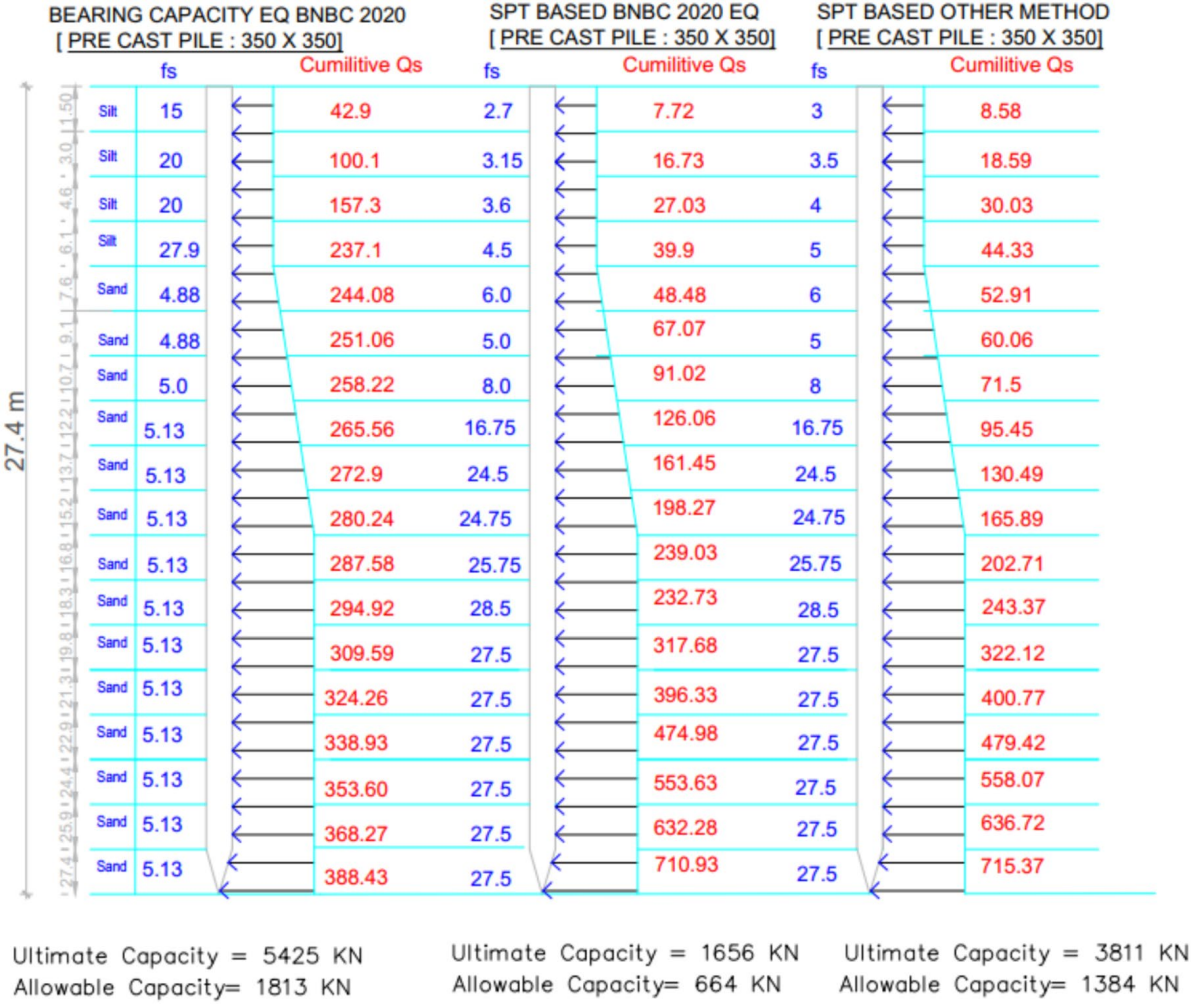
Variation of theoretical capacity in three methods for Project 6.

The subsoil profile for Project 6 comprises alternating layers of sand and silt. Variations in skin friction derived from the three theoretical methods are illustrated in Fig. 23, while Table 8 compares the predicted and measured pile capacities. The pile load test confirmed an allowable capacity of 559.0 kN. The SPT-based BNBC 2020 estimated 664.8 kN, exceeding the test value by 18.93%. The SPT-based other methods calculated a significantly higher capacity of 1384.7 kN, corresponding to a deviation of + 147.72%. The bearing capacity equation BNBC 2020 further overestimated the capacity at 1813.6 kN, with a notable deviation of + 224.45%.

**Table 8:** Statistical comparison of theoretical and load-test capacities for Project 6.

Method	Soil type	Cum. skin friction (kN)	Skin friction, *Q*_*s*_ (kN)	End bearing, *Q*_*b*_ (kN)	Allowable capacity (kN)	Difference with load test (559.0 kN)	Bias factor, λ = Pred/Test	Statistical summary
SPT-based BNBC 2020	Silt	39.9	710.93	1132	664.8	+ 18.93%	1.19	MAPE = 130.36%, Mean bias, λ̄ = 2.30 & COV = 0.37
	Sand	671.03						
SPT-based Other	Silt	44.33	715.37	3282.8	1384.7	+ 147.72%	2.48	
	Sand	671.04						
BCE BNBC 2020	Silt	237.1	388.43	5223.50	1813.6	+ 224.45%	3.24	
	Sand	151.33						

**Fig. 24:**
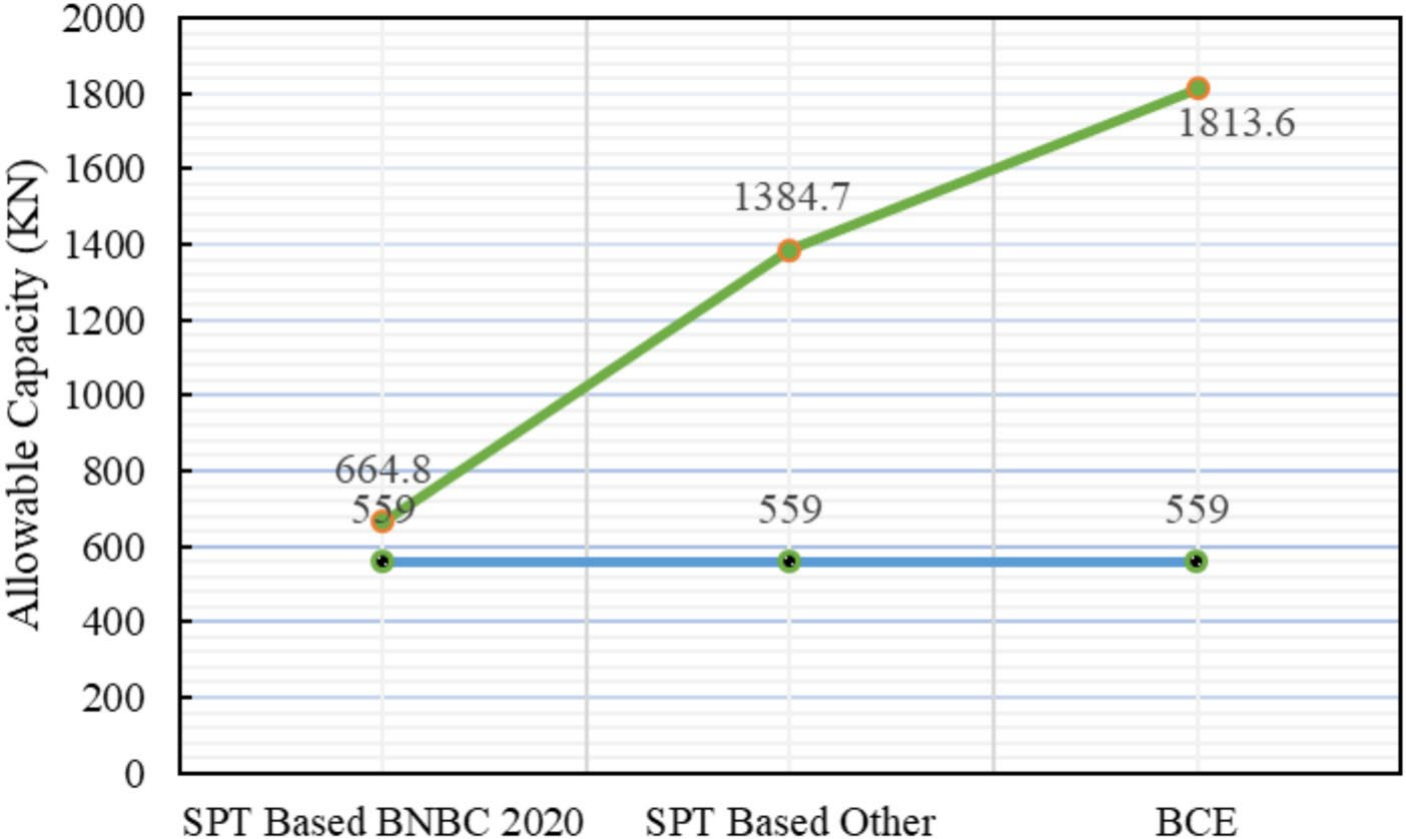
Deviations between predicted and actual pile capacities for Project 6.

Statistical analysis reveals that a mean bias (λ̄) of 2.30, a COV of 0.37, and a MAPE of 130.36% indicate comparatively moderate difference among the evaluated methods. In contrast, both the SPT-based other methods and the bearing capacity equation BNBC 2020 exhibited considerably higher bias factors, reflecting systematic overestimation of the actual pile capacity. These findings indicate that among the theoretical approaches, the SPT-based BNBC 2020 provides more reasonable estimates for piles embedded in sand and silt layers (Fig. 24**)**.

### Findings on suitable methods for pile capacity estimation

The selection of appropriate methods for estimating pile capacity depends on both soil and pile type. Statistical analysis across six projects indicates that the SPT-based BNBC 2020 method performs most consistently in sandy and silt–sand layered soils, whereas the SPT-based other methods (Meyerhof, 1976; Shioi & Fukui, 1982) shows better agreement in cohesive and clay-dominant conditions. The bearing capacity equation BNBC 2020 exhibited greater variability, tending to underestimate capacities in cohesive layers and overestimate them in granular soils.

Across all datasets, the mean bias (λ̄) ranged from 0.61 to 2.30, the COV from 0.05 to 0.44, and the MAPE from 22 to 130%, indicating that predictive accuracy is highly dependent on soil behavior. The SPT-based BNBC 2020 method maintained relatively low error indices (MAPE < 40%, COV < 0.35) in granular or mixed soils, while the SPT-based other methods provided better reliability in cohesive profiles with moderate variability (COV ≈ 0.30–0.40).

Tables 9, 10, 11 present a comparative analysis of the most suitable equations for skin friction, end bearing, and overall pile capacity, summarizing the effectiveness of the three theoretical methods under various soil conditions.

**Table 9:** Findings for skin friction.

Soil Layer	Precast Pile	Cast in Situ Pile	Comments	Ref. Project
Clay Layer	SPT based Other Method [after Shioi Fukui (1982)]	Same as Precast Pile	More accurate	Project—4
Sand Layer	SPT Based BNBC 2020 Method Bearing Capacity Equation BNBC 2020 [β –method]	Same as Precast Pile	Reasonable	Project -3
Layered Soil (Clay & Silt) Or (Clay & Sand)	Clay Layer [Layered Soil] Other Method [after Shioi Fukui (1982)]	Same as Precast Pile	Effective	Project -1
	Sand Layer [Layered Soil] Other Method [after Meyerhof (1976)]	Same as Precast Pile	Effective	Project -1
	Silt Layer [Layered Soil] Other Method [after Meyerhof (1976)]	Same as Precast Pile	Effective	-
Layered Soil (Silt & Sand)	Silt Layer [Layered Soil] SPT Based BNBC 2020 Method	Same as Precast Pile	Reasonable	Project -2 Project -5 Project -6
	Sand Layer [Layered Soil] SPT Based BNBC 2020 Method	Same as Precast Pile	Reasonable	Project -2 Project -5 Project -6

**Table 10:** Findings for end bearing.

Soil Layer	Precast Pile	Cast in Situ Pile	Comments	Ref. Project
Clay Layer	SPT-based Other Method [after Meyerhof (1976)]	Same as Precast Pile	More accurate	Project- 4
Sand Layer	SPT Based BNBC 2020 Method Bearing Capacity Equation BNBC 2020 [β –method]	Same as Precast Pile	Reasonable	Project -3
Layered Soil (Clay & Silt) Or (Clay & Sand)	Clay Layer [Layered Soil] Other Method [after Meyerhof (1976)]	Same as Precast Pile	Effective	Project -1
	Sand Layer [Layered Soil] Other Method [after Meyerhof (1976)]	Same as Precast Pile	Effective	Project -1
	Silt Layer [Layered Soil] Other Method [after Meyerhof (1976)]	Same as Precast Pile	Effective	-
Layered Soil (Silt & Sand)	Silt Layer [Layered Soil] SPT Based BNBC 2020 Method	Same as Precast Pile	Reasonable	Project -2 Project -5 Project -6
	Sand Layer [Layered Soil] SPT Based BNBC 2020 Method	Same as Precast Pile	Reasonable	Project -2 Project -5 Project -6

**Table 11:** Findings for overall pile capacity.

Soil Layer	Precast Pile	Cast in Situ Pile	Comments	Ref. Project
Clay Layer	SPT-based Other Method [Shioi Fukui (1982) and Meyerhof (1976)]	Same as Precast Pile	More Accurate	Project -4
Sand Layer	SPT-based BNBC 2020 & BCE BNBC 2020	Same as Precast Pile	Reasonable	Project -3
Layered Soil (Clay & Silt) Or (Clay & Sand)	SPT-based Other Method [Shioi Fukui (1982) and Meyerhof (1976)]	Same as Precast Pile	Effective	Project -1
Layered Soil (Silt & Sand)	SPT Based BNBC 2020 Method	Same as Precast Pile	Reasonable	Project -2 Project -5 Project -6

## Conclusion and recommendation

This study analyzed subsoil investigation reports and pile load test data from six projects in Bangladesh, including both precast and cast-in-situ piles. Findings indicate that:

1. In sandy soil, the SPT-based equations from BNBC 2020 provided reasonable results with pile load test results for determining skin friction and end bearing.
2. In clay soil, the SPT-based other methods from literature, Shioi & Fukui’s (1982) equation provided more accurate results for skin friction, while Meyerhof’s (1976) equation performed better for end bearing, compared to pile load test.
3. For cohesive or cohesionless or for layered soil, bearing capacity equations from BNBC 2020 showed reasonable outcomes with pile load test results for both skin friction and end bearing.
4. For layered soil containing clay, the Shioi & Fukui (1982) equation proved effective for determining skin friction in clay.
5. Meyerhof (1976) was suitable for calculating skin friction in sand and silt, as well as for end bearing. Notably, BNBC 2020 provided results for sand and silt that were nearly equivalent to those of Meyerhof (1976).
6. In layered soil without clay, SPT-based equations from BNBC 2020 displayed reasonable results for skin friction and end bearing when compared to pile load tests.
7. The findings indicate that in clay soil, the Shioi & Fukui (1982) SPT-based equation, which is not included in BNBC 2020, provided more accurate results than the current BNBC SPT-based correlations. Therefore, future revisions of BNBC may consider incorporating selected SPT-based methods from the literature. Specifically, Shioi & Fukui (1982) for skin friction and Meyerhof (1976) for end bearing are recommended for improved accuracy in clayey profiles.

This study does not propose a new pile-capacity equation. Instead, it provides a BNBC-focused evaluation of commonly used prediction and interpretation approaches using full-scale static pile load tests from Bangladesh projects, together with a mechanism-based explanation for soil-dependent agreement between measured and predicted capacities. A limitation is that CPT records were not consistently available in the compiled project archives; therefore, calculations focus on BNBC 2020 SPT-based procedures, while CPT is discussed for context and as a direction for database expansion. Future work should include CPT-supported sites so that direct CPT and CPTu methods can be evaluated alongside BNBC provisions.

Overall, these findings support targeted refinement of BNBC provisions while maintaining appropriate conservatism. BNBC 2020 is based on extensive national and international data, ensuring conservative treatment of soil variability. The present recommendations, particularly the inclusion of Shioi & Fukui (1982) and Meyerhof (1976) for clayey soils, refine this framework to improve predictive reliability under variable soil conditions while upholding code safety. This research provides a statistically supported basis for future calibration of BNBC pile design methods.

## Data Availability

All data collected during this study are included in this article and its related files (Soil Test data and Pile Load Test Data).

## List of symbols

*A*: Cross sectional area of pile
*A*_*b*_: End bearing area of pile
*A*_*s*_: Skin friction area (perimeter area) of pile
*D*: Diameter of pile
*C*: Apparent cohesion of soil
*C*_*u*_: Undrained cohesion of soil
*D*_*c*_: Critical depth of soil layer
*FS*: Factor of safety
*L*: Length of pile
*N*: Standard penetration test (SPT)
*N*_*60*_: Corrected SPT value for overburden pressure
*N*_*c*_*, N*_*q*_: Bearing capacity factors
*Q*_*b*_: End bearing at the base or tip of the pile
*Q*_*P*_: Load transferred to the soil at pile tip level
*Q*_*s*_: Skin friction or shaft friction or side shear
*Q*_*ult*_: Ultimate bearing/load carrying capacity
*Q*_*allow*_: Allowable or working axial load
*W*: Weight of the pile
*f*_*b*_: End bearing resistance on unit tip area of pile
*fs*: Skin frictional resistance on unit surface area of pile
*q*_*u*_: Unconfined compressive strength
*α*: Adhesion factor
*β*: Friction factor due to overburden
*σ*_*0*_: Initial effective stress at midpoint of a soil layer
*σ*_*v*_: Total vertical stress
*ϕ*: Apparent angle of internal friction
*ϕ**’*: Effective/drained angle of internal friction
